# A recyclable method for titanium extraction and oxygen evolution from Ti−bearing slags

**DOI:** 10.1016/j.fmre.2022.12.010

**Published:** 2022-12-27

**Authors:** Zhenghao Pu, Wei Wang, Zhe Wang, Mingyin Kou, Yiwa Luo, Jianbang Ge, Xin Tao, Mingyong Wang, Shuqiang Jiao

**Affiliations:** a State Key Laboratory of Advanced Metallurgy, University of Science and Technology Beijing, Beijing 100083, China; b School of Metallurgical and Ecological Engineering, University of Science and Technology Beijing, Beijing 100083, China; c Southern Marine Science and Engineering Guangdong Laboratory (Zhuhai), Zhuhai 519080, China

**Keywords:** Titanium, Molten oxide electrolysis, Inert anode, Liquid cathode, Depolarization

## Abstract

Despite its existence for more than 80 years, the titanium industry is still challenged by massive carbon emissions, high production costs, and large resource waste. More than one hundred million tons of Ti−bearing blast furnace slag (TB−slag) has been discarded in China because of the difficulty of reutilization, which requires efficient titanium extraction and recovery technologies. This paper describes a low−cost, carbon−emission−free method for Ti extraction and oxygen evolution via molten oxide electrolysis (MOE) vacuum distillation. After a comprehensive analysis of the binding energies and activities of liquid metals, the highlights of our study are as follows. 1) Sb has the best preferential deposition of Ti among a series of high−Ti−affinitive liquid metal cathodes (Cu, Ni, Pb, Sn, and Sb). 2) The Ir anode was first used in TB−slag with IrO_2_ formed on its surface to protect it from further corrosion. 3) An alloy containing Ti and Ca can be obtained by MOE, and Ti and Ca metals can be refined by further vacuum distillation. 4) A closed loop is formed in the overall process owing to the recyclable Sb cathode and continuous feeding of TB−slag into the electrolyte. This simple, low−cost, and environmentally friendly method can realize the efficient utilization of Ti resources and achieve carbon neutrality.

## Graphical abstract

We propose a molten oxide electrolysis (MOE)–vacuum distillation method based on special liquid cathodes and inert Ir anode to obtain Ti metal and O_2_ directly from cost–free Ti–bearing slags.

**Figure:**
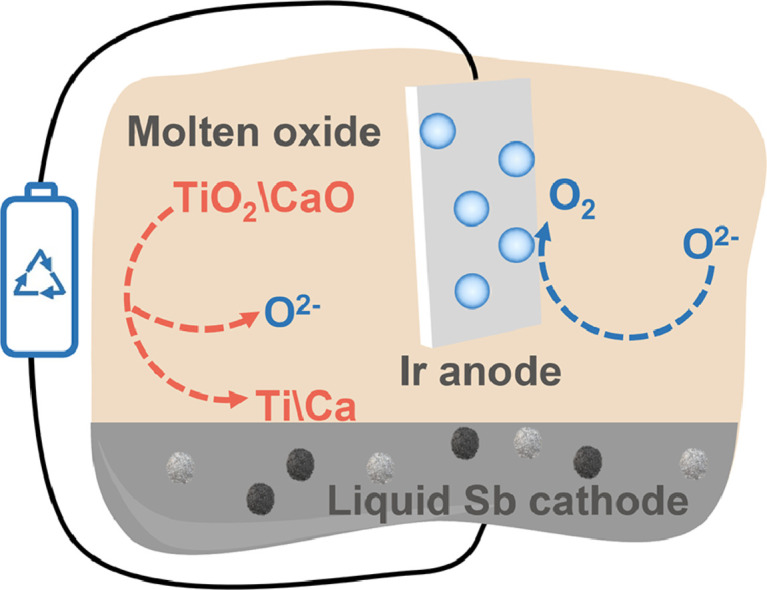

## 1 Introduction

Titanium (Ti) is one of the most important metals on Earth; it is widely used in the aerospace, marine, and biomedicine fields owing to its corrosion resistance, low density, and high strength. Since the 1940s, the Kroll process has been the primary industrial method for Ti production [1], [2], [3]. Nevertheless, evident drawbacks limit its large−scale application in industry. On the one hand, large carbon emissions and the use of chlorine gas lead to serious environmental problems, which is contrary to the theme of carbon neutrality. On the other hand, it is difficult to realize a massive price cut for Ti because of the long process flow and the high−cost Ti feedstocks. Therefore, exploring new low−cost Ti smelting technologies that are environmentally friendly and require shorter processes is urgently needed.

Owing to the appearance of the Hall–Héroult cells, the production of Ti using an efficient molten salt method has attracted increasing attention [4], [5], [6]. In the reported production processes, Ti feedstocks can be used as anodes, cathodes, or electrolytes for extracting Ti. In terms of cost, the use of Ti feedstocks as electrolytes is the best option because this approach can avoid the additional machining processes of anodes and cathodes by directly feeding electrolytes into the furnace. Therefore, since the 1950s, researchers have used Ti compounds such as TiO_2_, TiCl*_x_*, or K_2_TiF_6_ formulated into halides to obtain Ti by direct electrolysis. However, none of these compounds can be applied industrially owing to cost and efficiency problems [7], [8], [9], [10], [11]. In 1991, Sadoway et al. invented molten oxide electrolysis (MOE), which has become popular because of its low cost and environmental advantages [12]. Michel et al. and our group subsequently used industrial Ti oxides (TiO_2_−SiO_2_−MgO−Al_2_O_3_−CaO) as feedstocks for electrolysis and achieved the codeposition of Ti and silicon (Si) [12], [13], [14], [15]. Although depositing Ti alone is impossible, this study sheds new light on the direct extraction of Ti from industrial feedstocks.

Despite the significant progress achieved in the application of MOE in the electrolysis of Ti−bearing oxides, several challenges still need to be addressed, particularly the choice of suitable electrodes [16], [17], [18]. The use of conventional solid−state or low−activity liquid cathodes can result in Si deposition. We previously used high−activity liquid copper (Cu) cathodes to selectively deposit Ti without Si; however, Si was still co−deposited when the TiO_2_ in the electrolyte was reduced to a certain amount [19]. Therefore, an ideal liquid cathode with a stronger binding ability toward Ti and weaker binding ability toward Si is urgently needed. For anodes, the generally used graphite electrodes generate vast amounts of greenhouse gasses (mostly CO_2_), causing harm to the environment. Instead of generating CO_2_, O_2_ is produced if inert anodes are used in the electrolytes, thus effectively achieving carbon neutrality on a global scale [20], [21], [22]. In China, hundreds of millions of tons of Ti−bearing blast furnace slag (TB−slag) have been discarded as waste because of the difficulty of their direct utilization [23,24]. If these Ti−bearing slags are reused in MOE, environmental problems can be solved and significant economic value can be created.

**Fig. 1:**
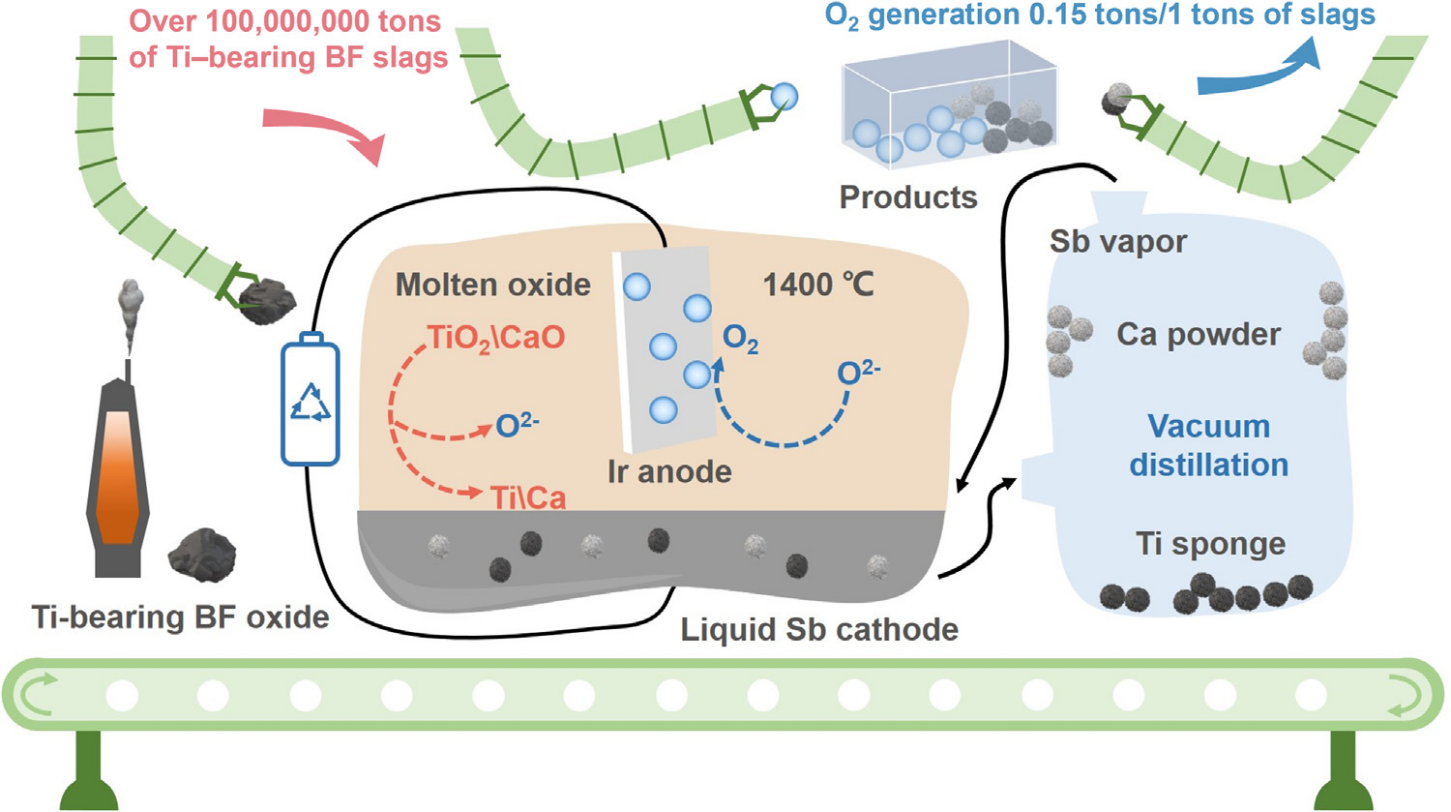
**Ti resource processing and smelting strategy**.

Herein, a recyclable and environmentally friendly method for electrolytic extraction of Ti and oxygen evolution based on a depolarized antimony (Sb) cathode and inert iridium (Ir) anode is proposed in this study (Fig. 1). TB−slag feedstock was used as the electrolyte for electrolysis, O_2_ was generated on the surface of the Ir anode, and Ti and calcium (Ca) metals were deposited in the liquid Sb cathode. The electrolyzed cathode was further refined to separate Ti, Ca, and recyclable Sb via vacuum distillation. The preferential deposition of Ti on the Sb cathode, corrosion mechanism of the Ir anode, and oxygen evolution process were studied through experiments, first−principles calculations, and thermodynamic analyses. As a promising low−carbon technology, the methodology proposed in this study can also be applied to the extraction of other metals from metal oxides.

## 2 Materials and methods

### 2.1 Materials

TiO_2_, CaO, Al_2_O_3_, MgO, SiO_2_, and CaF_2_ (99%) were purchased from Aladdin, and the Sb, nickel (Ni), tin (Sn), lead (Pb) and Ir were obtained from Jiaming Platinum Industry Co. Ltd. The contents of TB−slag (TiO_2_−SiO_2_−CaO−MgO−Al_2_O_3_) and TB−slag with added electroslag cosolvent (FTB−slag) (CaF_2_−TiO_2_−SiO_2_−CaO−MgO−Al_2_O_3_) are listed in Table S1. Before the electrolytic reaction, the metals and electrolytes were individually premelted in a resistance furnace under an argon (Ar) atmosphere for 2 h. The premelted samples were placed in a glove box during the experiments. To prepare the electrolytic reaction material, the premelted metal cathode and electrolyte were mixed well and heated to 1400 °C under Ar protection for direct current electrolysis, and the reaction process was temperature−corrected by a thermocouple located in the heating zone.

### 2.2 Electrochemical measurements

Electrochemical measurements were performed in an electrolytic cell at 1400 °C on a Princeton electrochemical workstation (P4000). Polarization curves of the TB−slag and FTB−slag were measured at a scan rate of 20 mV *s*^−1^ with molybdenum (Mo), Ir and tungsten (W) (3 mm in diameter) as working electrodes, Mo (3 mm in diameter) as the reference electrode, and graphite (10 mm in diameter) as the counter electrode. Moreover, CaF_2_−TiO_2_−CaO and FTB−slag were subjected to cyclic voltammetry (CV) measurements at a scan rate of 50 mV *s*^−1^ in the voltage range of −1.5 − 1 V, with Sb as the working electrode, Ir (3 mm in diameter) as the reference electrode, and graphite (10 mm diameter) as the counter electrode. The activity of the Sb alloy was measured using the electric potential method, and an electrolytic cell was constructed according to formulas 1 and 2. The open−circuit potential was measured for 2000 s.

(1) $$\left(\text{Ar}\right)\text{Mo}\left|\text{Cr}-Cr_{2}O_{3}\left(s\right)\right|\text{Zr}O_{2}\left(\text{MgO}\right)\left|\text{Sb}-\text{Ti}-O\left(\text{Melt}\right)\right|\left(\text{Ar}\right)\text{Mo}$$

(2) $$\left(\text{Ar}\right)\text{Mo}\left|\text{Cr}-Cr_{2}O_{3}\left(s\right)\right|\text{Zr}O_{2}\left(\text{MgO}\right)\left|\text{Sb}-\text{Ca}-O\left(\text{Melt}\right)\right|\left(\text{Ar}\right)\text{Mo}$$

### 2.3 Characterizations

The experimental procedure was performed using a direct−current power supply (PSM−3004, GW−INSTEK) for electrolysis. The basic properties of the anodes were investigated using thermogravimetric analysis−differential scanning calorimetry (TG−DSC, NETZSCH STA 2500). The properties of the anode gas during electrolysis were analyzed by continuous sampling gas analysis system (Hiden, HPR20). Synchrotron radiation (Beijing Synchrotron Radiation Facility, 4B9A beamline) and *X*−ray photoemission spectroscopy (XPS, Kratos AXIS Ultra DLD) were used to analyze the structural properties of the electrolytes after electrolysis. Scanning electron microscopy (SEM, GeminiSEM500) and an energy dispersive spectrometer probe (EDS, Thermo NS7) were used to analyze the morphology of the cathode and anode. The elemental content of the working electrode was analyzed by inductively coupled plasma emission spectrometry (ICP, ICPE−9810), and the cathode was described in detail by electron probe microanalysis (EPMA, 1720H) and industrial computed tomography (CT, YXLON FF35CT).

### 2.4 Modeling and simulations

All density function theory (DFT) calculations were performed using the Vienna Ab−initio simulation package (VASP) program. The energy cutoff (ENCUT) and *k*−point convergence should be calculated before performing any property calculations. The valence electronic states were extended in the plane−wave basis combination with an energy cutoff of 520 eV. During the structure optimization, the total energy change in the electronic structure partially self−consistent field (SCF) cycle was less than 10^−5^ eV and the residual force at the optimized atomic positions was less than 0.02 eV *Å*^−1^. The DFT functional was utilized at the Perdew−Burke−Ernzerhof (PBE) level. After obtaining the converged structure, the transition states and density of states were performed at a constant temperature of 0 K and the binding energy was performed at 293 K. The total number of simulation steps was 10,000 and the results were output at each step.

### 2.5 Technological calculation and evaluation

The CO/CO_2_ emissions (kg/t raw material) and raw material prices (USD/t) were evaluated for different Ti smelting methods. The carbon emission data of FFC (Eq. 3), MER (Eq. 4), USTB (Eq. 5), Kroll (Eq. 6) and QIT (Eq. 7) were calculated by using the principle of energy conservation at 100% efficiency.

(3) $$\text{Ti}O_{2}\;\left(1\;t\right)+C=\text{Ti}+CO_{2}\left(g\right)$$

(4) $$\text{Ti}O_{2}/C\;\left(1\;t\right)=\text{Ti}+CO_{2}\left(g\right)$$

(5) $$\text{Ti}C_{0.5}O_{0.5}\;\left(1\;t\right)=\text{Ti}+0.5\text{CO}\left(g\right)$$

(6) $$\text{Ti}O_{2}+C+Cl_{2}=\text{TiC}l_{4}\;\left(1\;t\right)+CO_{2}\left(g\right)$$

(7) $$\text{High}-\text{Ti}\;\text{slag}\;\left(\text{Ti}O_{2}=90\%\right)\;\left(1\;t\right)+C=\text{Ti}+CO_{2}\;\left(g\right)$$

Meanwhile, the prices of TiO_2_, TiO_2_/C, TiC_0.5_O_0.5_, TiCl_4_, and high−Ti slag (∼90% TiO_2_) were calculated based on the average price in China and the recent exchange rate. Among them, TiO_2_/C and TiC_0.5_O_0.5_ were calculated based on the amount of TiO_2_, graphite and high−Ti slag substances. It is worth noting that cost−free Ti−BF slag feedstock was used in this work, which does not produce carbon emissions and has byproducts of O_2_ and Ca.

## 3 Results and discussion

### 3.1 Application evaluation of Ir anode

**Fig. 2:**
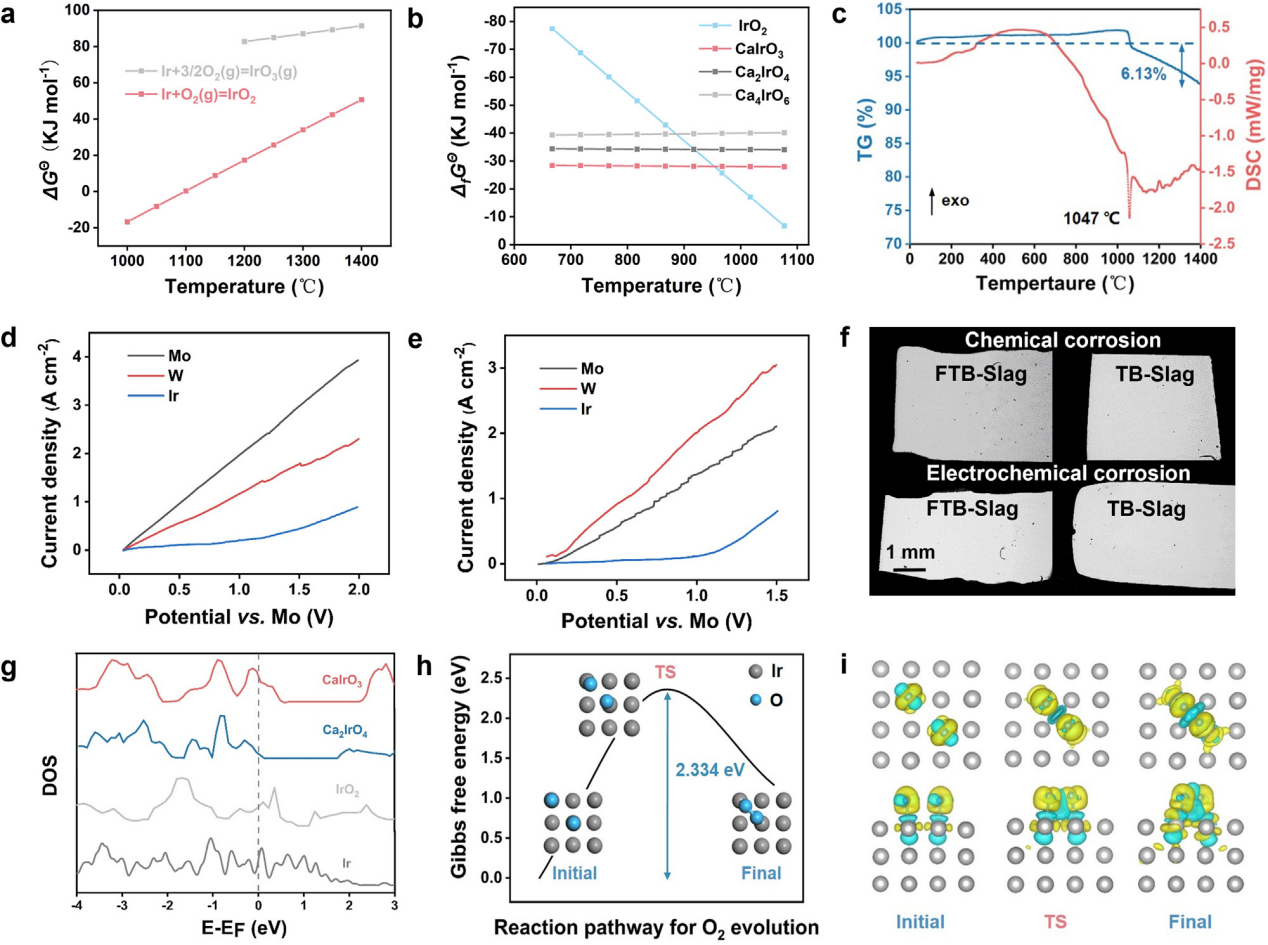
**Thermodynamic properties and anodic corrosion behavior of Ir**. (a) Relationship between Gibbs free energy and temperature for IrO_2_ and IrO_3_. (b) Thermodynamic properties of IrO_2_, CaIrO_3_, Ca_2_IrO_4_ and Ca_4_IrO_6_. (c) Thermogravimetric analysis−differential scanning calorimetry (TG−DSC) analysis of Ir from 25 to 1400 °C (air). (d) Polarization curve of the Ir anode in TB−Slag (20 mV *s*^−1^). (e) Polarization curve of Ir anode in FTB−Slag (20 mV *s*^−1^). (f) Scanning electron micrographs (SEM) of the chemical and electrochemical corrosion (1.5 A cm^−2^) of Ir in TB−Slag and FTB−Slag for 4 h (1400 °C). (g) DOS of Ir, IrO_2_, CaIrO_3_ and Ca_2_IrO_4_. (h) Calculation of the Gibbs free energy change of O_2_ evolution at the Ir interfacial. (i) Differential charge density of O_2_ evolution at the Ir interface.

The use of inert anodes is key to improving this carbon−intensive industry by reducing CO_2_ emissions, and the development of inert anode materials in MOE has been a focus of research. Ir is one of the most effective inert anodes for extracting iron (Fe) from iron ores (Fe_2_O_3_−SiO_2_−CaO−MgO−Al_2_O_3_); however, the stability of Ir is also challenged by the strong corrosive nature of oxides in high−temperature environments [11]. Thermodynamic calculations indicate that Ir exists as both IrO_2_ and IrO_3_ (Ir_2_O_3_ is unstable at high temperatures), and with solid IrO_2_ being the more stable between the two forms (Fig. 2a) [25]. On the other hand, Ir combines with CaO easily under high−temperature conditions to produce a series of compounds, *e.g.*, CaIrO_3_, Ca_2_IrO_4_, or Ca_4_IrO_6_; however, these compounds spontaneously decompose into Ir and O_2_ in the range of 1000 °C to 1400 °C (Fig. 2b). Therefore, a series of oxidation and decomposition reactions can occur at the Ir interface in the oxide electrolyte until an equilibrium state is reached [26,27].

Ir and IrO_2_ under Ar were analyzed by thermogravimetry–differential scanning calorimetry (TG−DSC) from 25 °C to 1400 °C. Ir exhibited good stability with only ∼1% mass increase below 1000 °C in air that results from Ir oxidation (Fig. 2c). When the temperature exceeded 1000 °C, the mass of Ir increased gradually by ∼3% and exhibited a significant decrease that started at 1047 °C and lasted until 1400 °C, resulting in a total mass loss of 6.13%. This trend was observed because Ir was initially oxidized to IrO_2_ upon heating in air; as the temperature continued to rise, IrO_2_ continued to be oxidized to gaseous IrO_3_ (heat absorption), resulting in mass loss. Meanwhile, the TG−DSC in Ar proved that IrO_2_ underwent a complete decomposition reaction at ∼930 °C [25]. After decomposition, Ir was stable in Ar without any changes. Therefore, Ir anodes must be used for inert gas protection at high temperatures.

To verify the stability of Ir in slags with different alkalinities, anodic polarization curves were obtained for Ir, Mo, and W electrodes (3 mm in diameter) in TB−slag and FTB−slag at 1400 °C (Fig. 2d,e; Fig. S1,S2). Mo and W were directly dissolved electrochemically in both slags, indicating that they are not applicable as inert anodes in the electrolytic process. Ir was electrochemically dissolved with a significant Faraday current at 1.3 V (*vs.* Mo) in TB−slag. For FTB−slag, the electrochemical dissolution of Ir starts at 1.1 V (*vs.* Mo), and the dissolution effect is more apparent than that in TB−slag because of the higher alkalinity. These results indicate that Ir is more stable in low−alkalinity slags.

To provide an intuitive proof, chemical and electrochemical corrosion of TB−slag and FTB−slag were tested at 1400 °C for 4 h (1.5 A cm^−2^) (Fig. 2f). The scanning electron microscopy (SEM) images of the chemical corrosion of naturally cooled Ir suggested a more notable corrosion effect on the FTB−slag, which exhibited a thickness loss of approximately 50 μm; by comparison, the corrosion effect of Ir on the TB−slag was not obvious, and no significant corrosion was observed at the cross−section (Fig. S3). The chemical corrosion results also prove the previous conjecture that the corrosion of Ir is more pronounced in slags with higher alkalinity. Simultaneously, a ∼20 μm corrosion layer was observed on the Ir anodes in both slags consisting of ∼500 nm Ir and tiny CaIrO_3_ or Ca_2_IrO_4_ particles; this layer was possibly formed due to the decomposition of both IrO_2_ and CaIrO_3_ during the cooling process or uneven chemical corrosion of oxides (Fig. S3−S5). Combining with the thermodynamic results, a conclusion can be formed that Ir forms protective layers of IrO_2_ at the interface in both electrolytes, but that IrO_2_ is eroded owing to the presence of CaO. The chemical corrosion rates of Ir in TB−slag and FTB−slag were calculated to be 0.07 (0.11 g *h*^−1^) and 0.14 mm *h*^−1^ (0.22 g *h*^−1^), respectively. Ir showed electrochemical corrosion result (1.5 A cm^−2^) similar to the chemical corrosion results, whereas the Ca−rich phase was almost absent at uniform corrosion rates in the TB−slag. The corrosion effect of Ir in the FTB−slag was more apparent. However, in contrast to the chemical corrosion results, the oxide layer on the Ir surface during electrochemical corrosion was not continuous and existed only in partial regions mainly because of the inhomogeneity of electrochemical corrosion on the Ir surface and the fast mass transfer rate under an electric field. The corrosion rates of Ir in TB−slag and FTB−slag under this condition were 0.13 (0.20 g *h*^−1^) and 0.17 mm^−1^ (0.26 g *h*^−1^), respectively, which are both significantly larger than the chemical corrosion rates. The density of states (DOS) of Ir, IrO_2_, CaIrO_3_, and Ca_2_IrO_4_, which could exist on the surface of Ir, was calculated (Fig. 2g). Ir and IrO_2_ have continuous energy bands at the Fermi energy level, whereas CaIrO_3_ and Ca_2_IrO_4_ have larger band gaps; these results indicate that Ir and IrO_2_ have better conductivity.

The oxygen evolution reaction (OER) at the interface of Ir (0 0 1) in the oxide system was calculated using first−principles calculations [28]. First, a transition state search for oxygen (O) atoms on the surface of Ir (0 0 1) was set up (Fig. S6). The initial position of the O atoms was above the interstitial void, fixing Ir in the cell; then, the O atoms gradually migrated toward Ir atoms with a migration energy barrier of 0.763 eV. Furthermore, the diffusion path of the O atoms and the reaction path of O_2_ evolution on the surface of the Ir anode were calculated (Fig. 2h). The stable−state O atoms approached each other and then bonded into O_2_, which was produced with an energy barrier of 2.334 eV. The differential charge density results reveal that when O atoms approach each other, their electron aggregation region (yellow) approaches, and no electron cloud exists in the intermediate electron depletion region (blue) (Fig. 2i). When the O atoms are bonded, the surrounding electron clouds become closer, and a common electron region appears in the intermediate electron depletion region. The results of the transition state search suggest that the rate−determining step in the O_2_ evolution process is the bonding of O atoms and that increasing the external electric field voltage can accelerate the O_2_ evolution reaction.

### 3.2 Properties of Ti−bearing slags

**Fig. 3:**
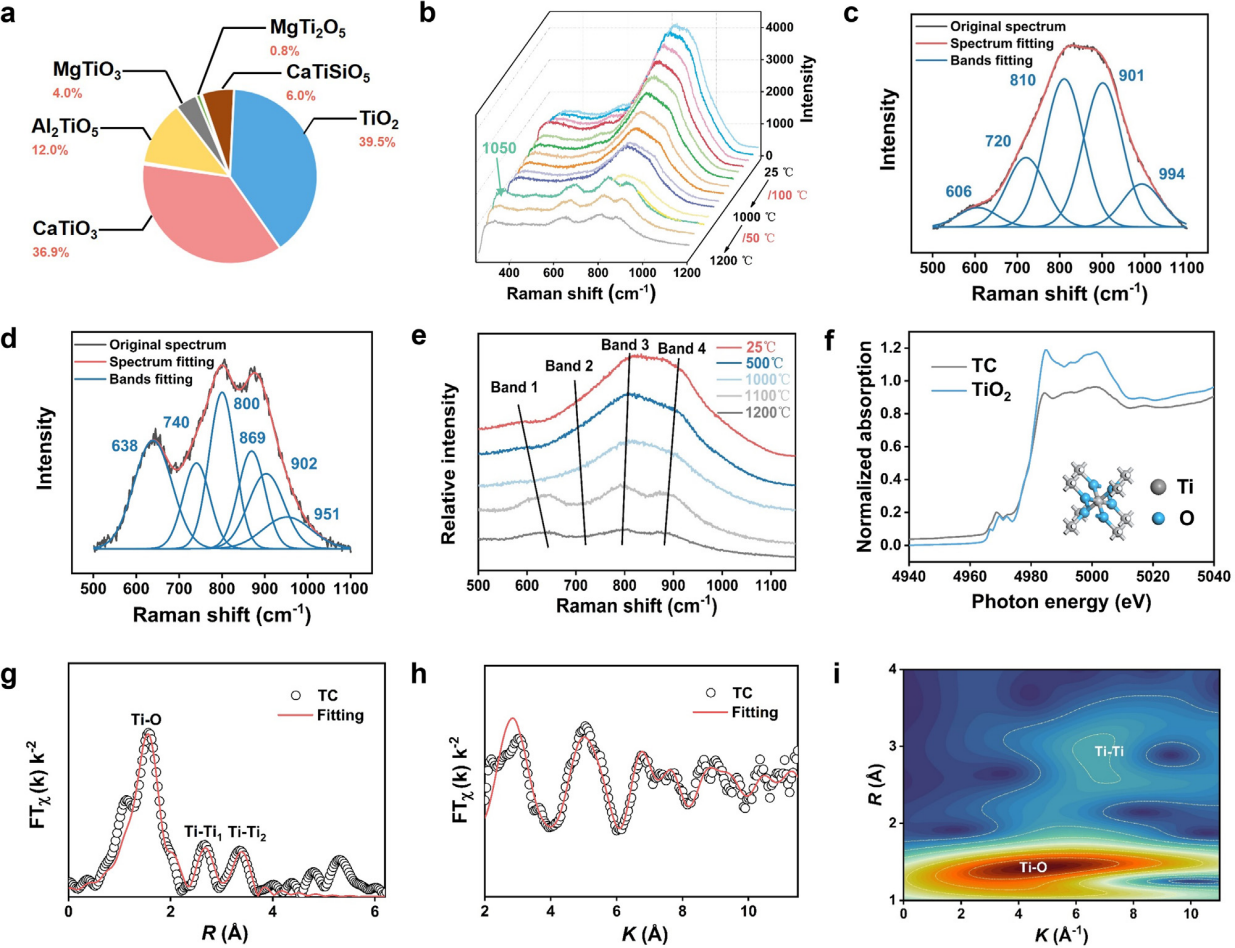
**Thermodynamic properties and structural characterization of Ti**−**bearing slag electrolytes**. (a) Statistics of Ti structural states in TB−Slag. (b) High−temperature Raman spectrum of TB−Slag quenched slag (25−1200 ℃). (c) Raman spectrum of TB−Slag quenched slag at 25 ℃. (d) Raman spectrum of TB−Slag quenched slag at 1200 ℃. (e) Raman spectrum analysis at several typical temperatures (25, 500, 1000, 1100 and 1200 ℃). (f) Synchrotron radiation full spectrum of TC and TiO_2_ standard samples. (g) *R*−space fit of TC. (h) *K*−space fit of TC. (i) Wavelet fitting analysis of TC.

The physicochemical characteristics of the electrolyte determine the mass−transfer rate of the electrolysis process. The ion–molecule coexistence theory was used to analyze the activities of each element in the mixtures of TB−slag at 1400 °C (Table S1) [29], [30], [31]. The amount of TiO_2_ fed in the TB−slag was less than that of SiO_2_, but its activity in the mixture was twice as high as that of SiO_2_. Because SiO_2_ is more acidic, it could preferentially combine with basic CaO and MgO and amphiphilic oxide Al_2_O_3_ in the mixture, thereby reducing the SiO_2_ activity. Therefore, a balance between the content of TiO_2_ and alkalinity must be achieved by adjusting the slag composition. Notably, the state of Ti in slags should be considered. The presence of three simple ions (Ca^2+^, Mg^2+^, and O^2−^), three simple molecules (Al_2_O_3_, SiO_2_, and TiO_2_) and 25 complex molecules in TB−slag indicates that the mass transfer processes of TB−slag become a black box (Tables S2,S3). Therefore, we focused on the states of Ti in this study. Nine states of Ti, mainly in the form of TiO_2_ and CaTiO_3_, were present in the slag, accounting for 76.4% of the total amount in the TB−slag. This indicates that TiO_2_ is weakly acidic in the slag and is more easily bonded to high−alkalinity CaO (Fig. 3a).

The structure of the quenched TB−slag and the coordination state of Ti analyzed by high−temperature Raman spectroscopy ranging from 25 °C to 1200 °C are shown in Fig. 3b. For TB−slag, three states existed during the heating process: glass, crystal, and melt. Notably, a crystal phase was evident at the beginning of 1050 °C; then, the peaks gradually broadened, and the melt became more disordered as TB−slag melting progressed. In the Raman spectra at 25 °C and 1200 °C, only the monomer (Q^0^), dimer (Q^1^), chain (Q^2^), and possibly sheet (Q^3^) were present in the high−frequency region (800–1200 cm^−1^) of silicate, and fully polymerized ${\text{SiO}}_{4}^{4-}$−tetrahedra (Q^4^) was not present (Fig. 3c,d) [32]. The vibrations in the mid−frequency region of ∼720 and ∼800 cm^−1^ and low−frequency region of 600−650 cm^−1^ were definitely related to Ti [33]. The correlation coefficients of the fitted curves with the original curves all exceeded 0.999.

Furthermore, the Raman spectra were plotted for five typical temperatures with four distinct bands in the region of 600−900 cm^−1^ (Fig. 3e). Band 1 (600−650 cm^−1^) represents the 6−coordinated Ti^4+^ stretching vibration of Ti−O, Band 2 (720 cm^−1^) represents the Ti−O or Ti/Si−O deformation in the chain or sheet, band 3 (∼790 cm^−1^) represents the vibration of Ti−O in ${\text{TiO}}_{4}^{4-}$ monomers, and band 4 (∼870 cm^−1^) represents the stretching vibration of Ti−O in the 5−coordinated Ti^4+^ or $Ti_{2}O_{6}^{4-}$ chain [34]. Although the coordination states of Ti in TB−slag have been observed, their specific contents cannot be determined. However, Ti preferentially acts as a network modifier when the TiO_2_ content in the melt exceeded 20%, which consequently required Ca^2+^ and Mg^2+^ to achieve charge equilibrium; thus, an appropriate alkalinity facilitated the formation of Ti monomers and dimers that promoted Ti deposition.

TiO_2_−CaO (TC), the most abundant component in TB−slag, was prepared to avoid interference from ineffective components, such as Si, Al, and Mg. The coordination state of Ti in TC was investigated using Ti *K*−edge *X*−ray absorption near−edge spectroscopy (XANES). The XANES spectra of TC obtained using TiO_2_ as the standard material are shown in Fig. 3f. The edge spectra of TiO_2_ exhibited three weak peaks and a distinct inflection. The duplex simple 3*d* orbitals of the antibonding state showed different symmetry from the 4*p* orbitals and no hybridization, and the 1*s*−3*d* leap remained blocked, leading to faint edge spectral peaks. Meanwhile, the edge spectra peak of TC exhibited a slightly higher broad peak, indicating that TC had a different coordination structure from that of standard TiO_2_. The TC and TiO_2_ absorption edges almost overlapped, indicating that they had the same valence. The lower intensity of the TC white−line peak was due to its weak structural symmetry. The XANES R−space and K−space fits of TC and TiO_2_ by Fourier transform revealed three bonds, namely, Ti−O, Ti−Ti_1_, and Ti−Ti_2_, with bond lengths of 1.94, 3.01 and 3.82 Å, which were close to those of the standard TiO_2_ sample of 1.93, 3.00 and 3.80 Å, respectively (Fig. 3g,h; Fig. S7, Table S4). Compared with those of TiO_2_, the smaller coordination numbers of Ti−O and Ti−Ti in TC indicate a more disordered structure, which makes the reduction of TiO_2_ in TC to Ti metal easier. The wavelet fitting results revealed that the bond length of Ti−O was smaller than that of Ti−Ti and that some Ti−Ti bonds were formed because of the presence of vacancies in TB−slag despite their much smaller number than that of Ti−O bonds (Fig. 3i). In summary, the Ti−O bond coordination number in TB−slag is lower than that in TiO_2_; thus, the reaction occurs at a faster rate, leading to enhanced current efficiency.

### 3.3 Application of liquid cathode in MOE

**Fig. 4:**
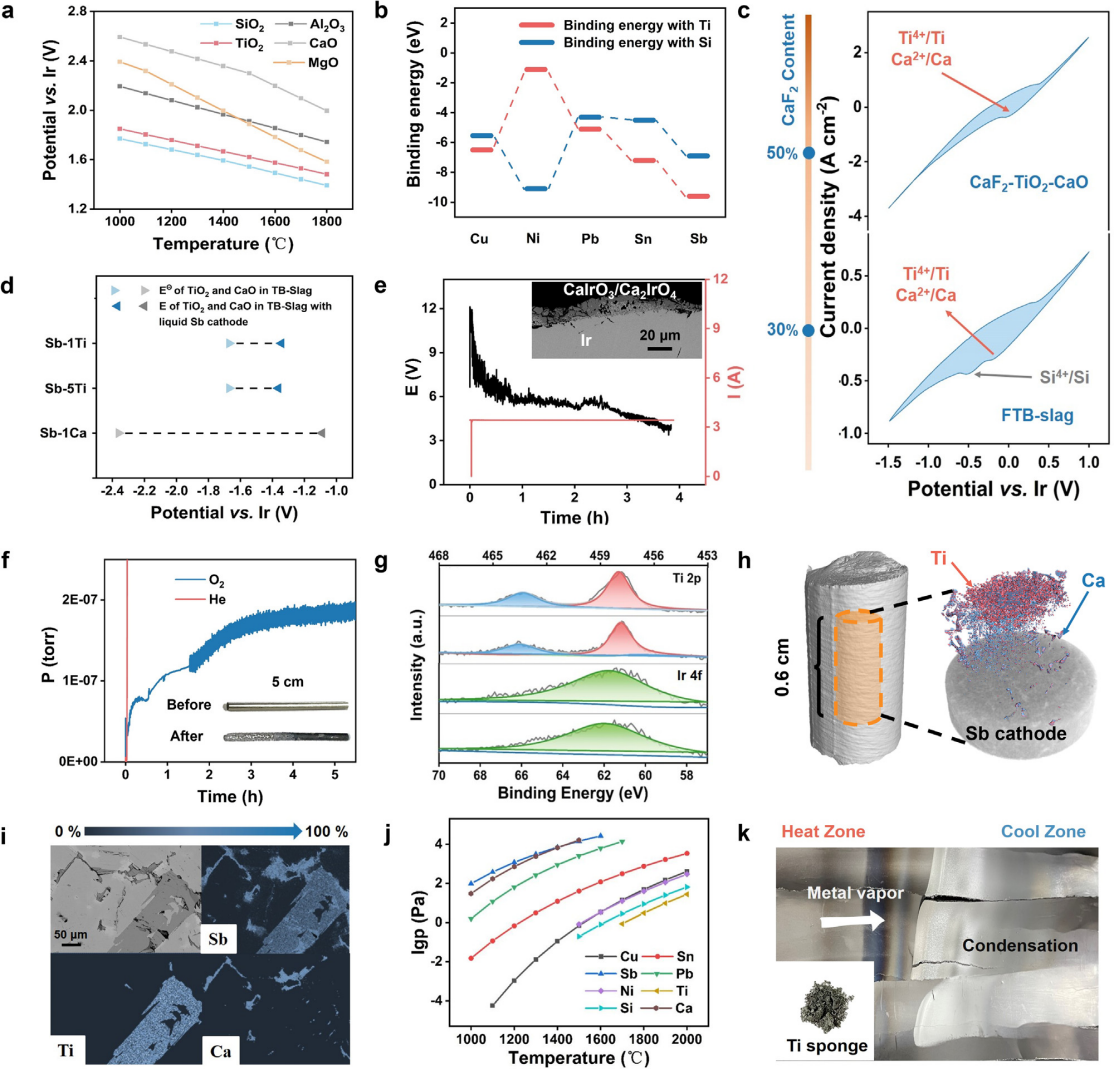
**Simulation and electrochemical behavior of liquid cathodes and characterization of electrolysis products**. (a) Ellingham diagram of TiO_2_, SiO_2_, Al_2_O_3_, MgO and CaO in TB−Slag. (b) Simulated binding energy calculations of Cu, Ni, Pb, Sn and Sb with Ti and Si. (c) Cyclic voltammetry curves of the Sb electrode in CaF_2_−TiO_2_−CaO and FTB−Slag (scan rate: 50 mV *s*^−1^, reference electrode: Ir, counter electrode: graphite). (d) TiO_2_ or CaO deposition potential calculation in TB−Slag (1400 ℃). (e) Electrolysis curves and Ir anode morphology (1.5 A cm^−2^). (f) Online gas monitoring analysis of the Sb cathode electrolysis process in TB–Slag (1400 ℃, 2.25 A cm^−2^). (g) XPS of Ti and Ir in TB−Slag after electrolysis (1400 ℃, 1.5, 2.25 A cm^−2^). (h) CT reconstruction analysis of the Sb cathode after electrolysis in TB−Slag (1400 ℃, 2.25 A cm^−2^). (i) EPMA of the Sb cathode after electrolysis in TB−Slag (1400 ℃, 2.25 A cm^−2^). (j) Metal saturation vapor pressure as a function of temperature. (k) Schematic diagram of the results of vacuum distillation of the Sb cathode.

It is difficult to directly extract Ti from TB−slag. In such electrolytes, SiO_2_ is preferentially reduced (Fig. 4a), thereby significantly limiting the deposition of Ti. Therefore, in such cells, the preferential deposition of Ti can only be achieved by optimizing the cathode side. In the following equation, *E* is the standard electrode potential, *T* is the reaction temperature, *R* and *F* are the gas and Faraday constants, respectively, $a_{O}$ is the activity of TiO_2_ in TB−slag, and $a_{R}$ is the activity of Ti in the cathode. To realize the preferential deposition of Ti in TB−slag, a liquid cathode with good affinity to Ti is needed to reduce the activity of Ti in the cathode and consequently accelerate the reaction kinetic rate.

(8) $$E=E^{0}-\frac{RT}{4F}\text{In}\frac{a_{R}}{a_{O}}$$

Given the basic properties of liquid cathodes, Cu, Ni, Pb, Sn, and Sb were selected as potential materials and their binding energies with Ti and Si were calculated (Fig. S8) [35]. Cu, Pb, Sn, and Sb exhibited good potential as liquid cathodes because of their stronger binding ability with Ti; notably, the binding energy of Ti@Sb was −9.56 eV, which was much stronger than that of other metals (Fig. 4b). Furthermore, experimental studies via galvanostatic electrolysis with Pb, Sn, Ni, and Sb cathodes were performed on TB−slag. All reactions were conducted at a cathodic current density of 0.1 A cm^−2^. After electrolysis, a large amount of Si was deposited on the Pb cathode, while the Ni cathode exhibited an extremely low current efficiency with Ti and Si codeposition (Fig. S9, S10). Both Sn and Sb cathodes were able to deposit Ti and inhibit Si deposition, and surprisingly, a large amount of Ca was also deposited on the Sb cathode (Fig. S11, S12). After considering the current efficiency factors, Sb was identified as an ideal cathode material for TB−slag electrolysis.

CV measurements were performed on CaF_2_−TiO_2_−CaO and FTB−slag, which had higher conductivities than TB−slag (reference electrode: Ir; counter electrode: graphite; 50 mV *s*^−1^, Fig. 4c), with Sb as the working electrode. Scanning from the open−circuit potential in the negative direction, a clear Faraday current and reduction peaks appeared starting at 0 V (*vs.* Ir) in CaF_2_−TiO_2_−CaO. When the scanning direction was switched to the positive direction, a relative oxidation peak was observed at 0.2 V (*vs.* Ir). In combination with the Sb cathode electrolysis results, the reduction peak at 0 V (*vs.* Ir) corresponds to the codeposition of Ti^4+^ and Ca^2+^. Working electrode Sb exhibited two distinct reduction peaks in the CV of the FTB−slag. In combination with the results of CaF_2_−TiO_2_−CaO CV, the reduction peak at −0.2 (*vs.* Ir) was attributed to the codeposition of Ti^4+^ and Ca^2+^, whereas that at −0.5 (*vs.* Ir) was ascribed to the deposition of Si^4+^. Similarly, only one oxidation peak of 0.2 V (*vs.* Ir) was observed in FTB−slag, which is due to the slow mass transfer of the oxide and insignificant oxidation reaction. However, the conductivity of the electrolyte was significantly enhanced when the CaF_2_ content was 50%.

To explain the depolarization deposition mechanism of Ti and Ca on the Sb cathode, the activities of Ti and Ca in Sb−1Ti, Sb−5Ti, and Sb−1Ca were measured using the electromotive force method (Fig. S13, Table S5) [36], [37], [38]. The activity of Ca in Sb−1Ca was only 2 × 10^−9^, whereas the activity of Ti in Sb−1Ti was 1.15 × 10^−5^, indicating the extreme affinity of Sb and Ca. The deposition potentials of Ti and Ca in the corresponding Sb cathodes were obtained by combining the results of previous activity calculations for each component of the TB−slag (Fig. 4d). The results reveal that Ca and Ti achieve positive potential shifts of ∼1.2 and 0.3 V (*vs.* Ir), respectively, which agree well with the CV results showing that the Sb cathode can result in the depolarized deposition of Ti and Ca.

Galvanostatic electrolysis (0.75, 1.5, and 2.25 A cm^−2^ for 4 h at 1400 °C) was conducted on TB−slag with the preferred Sb as the cathode and Ir as the anode (Fig. S14). The cell voltage increased significantly at the beginning of electrolysis and then decreased slowly with reaction time at 2.25 A cm^−2^. This phenomenon was not observed at a current density of 1.5 A cm^−2^, indicating that the Ir anode interface had a greater tendency to form CaIrO_3_ and Ca_2_IrO_4_ at high current densities, leading to an increase in reaction−cell voltage (Fig. 4e). As the electrolysis reaction occurred, the decomposition and synthesis reactions of CaIrO_3_, Ca_2_IrO_4_, and IrO_2_ on the surface of the Ir anode gradually reached equilibrium, and the cell voltage decreased. As shown in the CV results (Fig. 4c), a higher cell voltage resulted in lower voltage efficiency during electrolysis. To enhance energy efficiency, the composition and conductivity of the electrolytes were optimized. The electrolysis reactions were measured using a continuous sampling gas analysis system. To ensure the reliability of the gas detection results, electrolysis was started after 1.5 h of sampling (Fig. S15). A notable O_2_ evolution signal was detected on the anode during the electrolytic reaction of TB−slag at 1.5 and 2.25 A cm^−2^ (Fig. 4f, Fig. S15). By contrast, no O_2_ evolution occurred at 0.75 A cm^−2^ in TB−slag, revealing that the reaction overpotential was insufficient for electrolysis to proceed under this condition. Apparent electrochemical corrosion at 2.25 A cm^−2^ was observed on the Ir anode (Fig. 4f).

Elemental analysis of the Sb cathode after electrolysis revealed that no deposition occurred inside the electrolyzed Sb cathode in TB−slag at 0.75 A cm^−2^ for 4 h; this result agreed with those of sampling gas analysis, that is, the overpotential was insufficient for driving electrolysis under these conditions (Table S6). As the electrolytic current density increased, the amounts of Ti and Ca deposited in the electrolytic cathode increased. Notably, 0.51 wt% Ti and 0.44 wt% Ca were distributed inside the electrolyzed Sb cathode at 2.25 A cm^−2^ in TB−slag with a cathodic efficiency of 18.96%; these results were much higher than those obtained under other reaction conditions. However, the deposition of Ti decreased because of the deposition of Ca. A reduction in the Ca^2+^ concentration in the electrolyte or the use of Sb−Ca cathodes can effectively inhibit Ca deposition. Notably, almost no Si deposition was observed on the Sb cathode under different experimental conditions. In general, the total contents of CaO and TiO_2_ in TB−slag were approximately 50%. Compared to molten salt electrolysis, the MOE method has a low current efficiency because of the high resistance and low conductivity of the oxides, which can decrease production efficiency. Furthermore, TB−slag after electrolysis at 1.5 and 2.25 A cm^−2^ was subjected to *X*−ray photoemission spectroscopy analysis, and only Ti^4+^ was found in the slags, indicating a rapid four−electron transfer process (Fig. 4g). In addition, tiny amounts of Ir^4+^ were detected in the electrolyzed slags, indicating the electrochemical dissolution of the anode.

The SEM image of the electrolyzed Sb cathode at 2.25 A cm^−2^ in TB−slag revealed the existence of regular precipitated phases with a size of ∼100 μm; moreover, the states of Ca were impossible to separate owing to the interference of the excited states of Ca and Sb (Fig. S16). Analysis using point scanning indicated that the Ti and Ca distributions were precipitated in the forms of TiSb_2_ and CaSb_2_, which had regular and shaped structures, respectively. Meanwhile, minimal Ir deposition was observed within the Sb cathode (Fig. S17). The same Sb cathode was reconstructed by industrial computed tomography on a large scale. The Ti−rich and Ca−rich phases tended to be distributed in the upper part of the Sb cathode with a more uniform distribution because of the edge effect of CT on the internal structure of the cathode for elemental species discrimination (Fig. 4h, Fig. S18) [39]. The volume percentages of the Ti−rich and Ca−rich phases were 3.76% and 4.03%, respectively, and the estimated cathode efficiency was 20.02%, which was close to that measured by inductively coupled plasma emission spectrometry above. The results of electron probe microanalysis revealed the morphology of Ti and Ca on the Sb cathode, which was consistent with the previous conjecture that Ti with a length of ∼300 μm precipitated regularly, whereas Ca was randomly distributed around the Ti−rich phases in a fine line (Fig. 4i). Coexistence of the three metals was not observed. In summary, in the electrolysis of TB−slag, the depolarization of the Sb cathode to Ti and Ca enables the deposition of Ti and Ca and suppresses the deposition of Si.

The electrolyzed cathodes must be further separated to obtain Ti metal. Vacuum distillation was used to separate the electrolyzed cathodes because of the large difference in saturated vapor pressures between Sb and Ti (Fig. 4j) [40,41]. Electrolyzed Sb cathodes (20 g, TB−slag, 2.25 A cm^−2^) were subjected to vacuum distillation at 1200 °C under 1 Pa for 4 h. The reaction was performed in a horizontal furnace, and the volatiles were collected using graphite paper (Fig. 4k). Because Sb and Ca had high vapor pressures, their metal vapors moved toward the cool zone and condensed, yielding a residue in the form of a Ti sponge with Ti content greater than 90%. Therefore, vacuum distillation can effectively separate Ti, and if the reactor is modified, the overall process can be operated continuously to obtain Ti and Ca products.

**Fig. 5:**
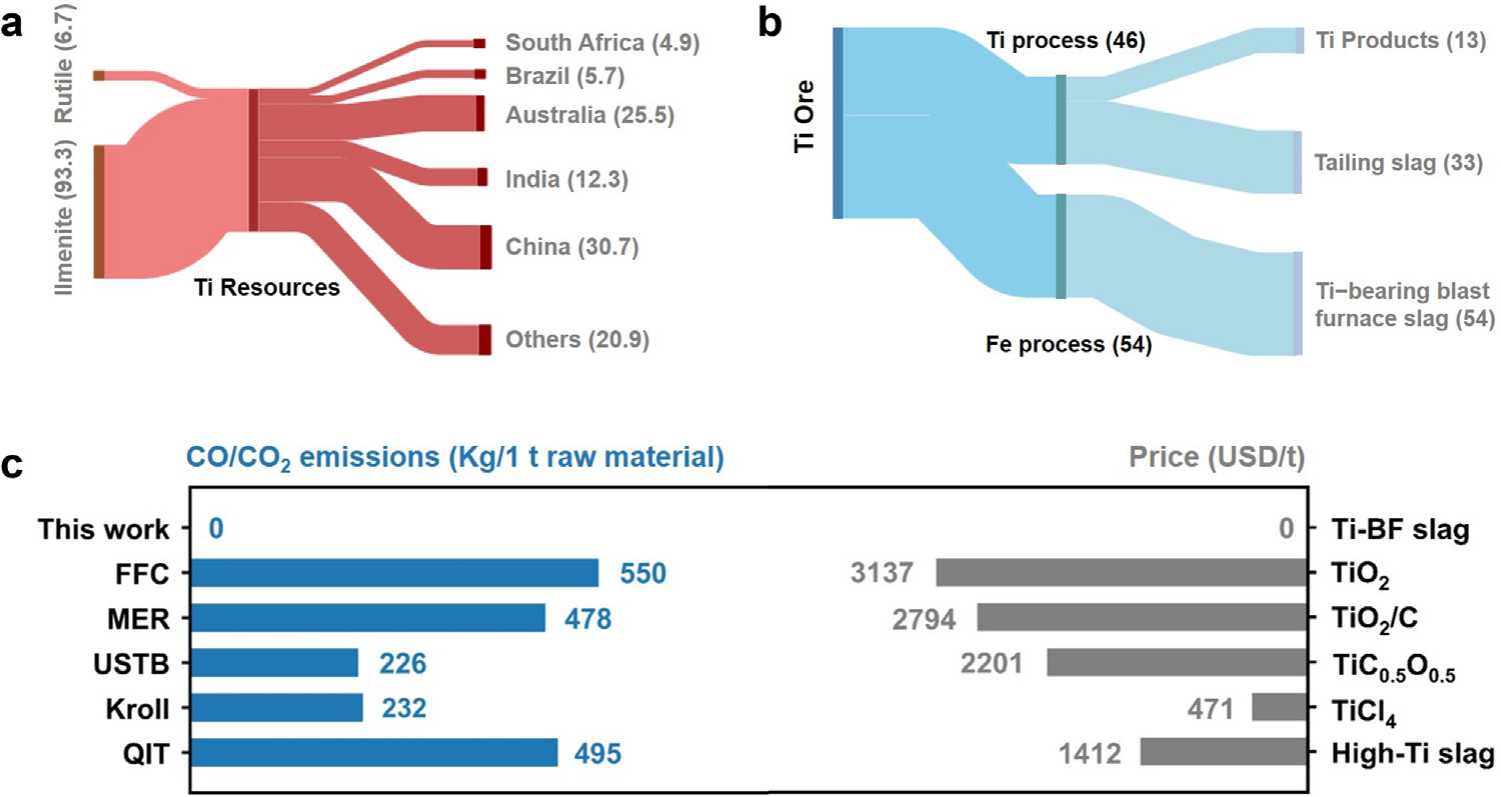
**Evaluation of Ti resources and smelting methods**. (a) Global distribution of Ti resources in 2021 (%). (b) Processing strategy of titanium ore in China (%). (c) Evaluation of Ti smelting methods (carbon emission and raw material price).

Ranking seventh among all metals, Ti is extremely abundant in the Earth's crust, with ilmenite and rutile being its main ore types. China holds 30.7% of the world's Ti reserves, virtually all of which exist as ilmenite (Fig. 5a) [42]. In the Chinese Ti industrial process, only 13% of the Ti resources are utilized, and approximately 54% of the Ti resources become waste TB−slag, which contains approximately 20% TiO_2_ and can also be used as a raw material for Ti production (Fig. 5b) [43], [44], [45]. The incomplete reduction reaction of TiO_2_ and C results in the high production cost of Ti. Meanwhile, the Ti industry is greatly challenged by severe environmental problems. In this work, cost−free raw materials and unique electrodes were employed in an electrolysis process that yielded no carbon emission and was superior to the previously reported FFC, MER, USTB, Kroll, and QIT methods (Fig. 5c), which output massive carbon emissions. The electrolytes and cathode can be replaced during electrolysis, thus enabling continuous production, and the valuable Ti, Ca, and O_2_ can be obtained simultaneously. However, considerable effort is required to reduce the energy consumption in the production process. Nevertheless, this work shows great application potential in the Ti industry and can be extended to the smelting processes of other metal oxides and oxygen production in space.

## 4 Conclusion

An advanced technology for extracting Ti from cost−free TB−slag or inexpensive Ti ores was proposed. After simple galvanostatic electrolysis and subsequent vacuum distillation, Ti metal was obtained with O_2_ and Ca as byproducts. Sb was preferred as a liquid cathode for the deposition of Ti and Ca in SiO_2_−bearing slags because of its depolarization effect. Owing to the use of the Ir anode, O_2_ instead of CO_2_ is generated, thereby promoting carbon neutrality. The MOE vacuum distillation method used in this work also has the potential to be applied to the extraction of metals such as Fe and Ni from their oxides.

## Declaration of competing interest

The authors declare that they have no conflicts of interest in this work.

## Appendix Supplementary materials

Image, application 1

## References

[1] Zhang D.Y., Qiu D., Gibson M.A. (2019). Additive manufacturing of ultrafine–grained high–strength titanium alloys. Nature.

[2] Zhao S.T., Zhang R.P., Yu Q. (2021). Cryoforged nanotwinned titanium with ultrahigh strength and ductility. Science.

[3] Kroll W.J. (1940). The Production of ductile titanium. Trans. Am. Electrochem. Soc..

[4] Wang K.L., Jiang K., Chung B. (2014). Lithium–antimony–lead liquid metal battery for grid–level energy storage. Nature.

[5] Zou X.L., Ji L., Ge J.B. (2019). Electrodeposition of crystalline silicon films from silicon dioxide for low–cost photovoltaic applications. Nat. Commun..

[6] Jiao H.D., Qu Z.L., Jiao S.Q. (2021). Quantificational 4D Visualization of Industrial Electrodeposition. Adv. Sci..

[7] Chen G.Z., Fray D.J., Farthing T.W. (2000). Direct electrochemical reduction of titanium dioxide to titanium in molten calcium chloride. Nature.

[8] Vuuren D.S.V., South J. (2009). A critical evaluation of processes to produce primary titanium. Afr. Inst. Min. Metall..

[9] Jiao S.Q., Zhu H.M. (2006). Novel metallurgical process for titanium production. J. Mater. Res..

[10] F. Cardarelli, U.S. Patent, 2009, 7504017.

[11] Allanore A. (2015). Features and challenges of molten oxide electrolytes for metal extraction. J. Electrochem. Soc..

[12] Sadoway D.R. (1991). The electrochemical processing of refractory metals. JOM.

[13] Jiao H.D., Tian D.H., Wang S. (2017). Direct preparation of titanium alloys from Ti–bearing blast furnace slag. J. Electrochem. Soc..

[14] Martin–Treceno S., Hughes T., Weaver N. (2021). Electrochemical study on the reduction of Si and Ti from molten TiO_2_–SiO_2_–Al_2_O_3_–MgO–CaO slag. J. Electrochem. Soc..

[15] Martin–Treceno S., Weaver N., Allanore A. (2020). Electrochemical behaviour of titanium–bearing slag relevant for molten oxide electrolysis. Electrochim. Acta.

[16] Jiao H.D., Tian D.H., Tu J.G. (2018). Production of Ti–Fe alloys via molten oxide electrolysis at a liquid iron cathode. RSC Adv..

[17] Fried N.A., Rhoads K.G., Sadoway D.R. (2001). Transference number measurements of TiO_2_–BaO melts by stepped–potential chronoamperometry. Electrochim. Acta..

[18] Takenaka T., Matsuo H., Sugawara M. (2010). High temperature electrolysis of Ti and its alloys with a DC–ESR unit. Key Eng. Mater..

[19] Pu Z.H., Jiao H.D., Mi Z.S. (2020). Selective extraction of titanium from Ti–bearing slag via the enhanced depolarization effect of liquid copper cathode. J. Energy Chem..

[20] Allanore A., Yin L., Sadoway D.R. (2013). A new anode material for oxygen evolution in molten oxide electrolysis. Nature.

[21] Esmaily M., Mortazavi A.N., Birbills N. (2020). Oxidation and electrical properties of chromium–iron alloys in a corrosive molten electrolyte environment. Sci. Rep..

[22] Kim H.J., Paramore J., Allanore A. (2010). Stability of iridium anode in molten oxide electrolysis for ironmaking: influence of slag basicity. ECS Trans.

[23] Cai Y.F., Song N.N., Yang Y.F. (2022). Recent progress of efficient utilization of titanium–bearing blast furnace slag. Int. J. Miner., Metall. Mater..

[24] M.Y He, Teng L.M., Gao Y.X. (2022). Simultaneous CO_2_ mineral sequestration and rutile beneficiation by using titanium−bearing blast furnace slag: Process description and optimization. Energy.

[25] Jacob K.T., Okabe T.H., Uda T. (1999). Solid–state cells with buffer electrodes for the measurement of thermodynamic properties of IrO_2_, CaIrO_3_, Ca_2_IrO_4_, Ca_4_IrO_6_. J. Electrochem. Soc..

[26] Mcdaniel C.L., Schneuder S.J. (1972). Phase relations in the CaO–IrO_2_–Ir system in air. J. Solid. State. Chem..

[27] Bell W.B., Tagami M. (1966). Study of gaseous oxides, chloride, and oxychloride of iridium. J. Phys. Chem..

[28] Klyukin K., Zagalskaya A., Alexandrov V. (2018). Ab initio thermodynamics of iridium surface oxidation and oxygen evolution reaction. J. Phys. Chem. C.

[29] Duan S.C., Guo X.L., Guo H.J. (2016). A manganese distribution prediction model for CaO–SiO_2_–FeO–MgO–MnO–Al_2_O_3_ slags based on IMCT. Ironmak. Steelmak..

[30] Shi C.B., Yang X.M., Jiao J.S. (2010). A sulphide capacity prediction model of CaO–SiO_2_–MgO–Al_2_O_3_ ironmaking slags based on the ion and molecule coexistence theory. ISIJ Int..

[31] Zhou Y., Zhu R., Wang H.Y. (2020). Effect of various components on the distribution of phosphorus in CaO–FeO–MgO–SiO_2_–MnO–TiO_2_–V_2_O_5_–P_2_O_5_ slag based on IMCT. Ironmak. Steelmak..

[32] You J.L., Jiang G.C., Xu K.D. (2001). High temperature Raman spectra of sodium disilicate crystal, glass and its liquid. J. Non–Cryst. Solids.

[33] Farges F., Brown G.E., Navrotsky A. (1996). Coordination chemistry of Ti (IV) in silicate glasses and melts: II. Glasses at ambient temperature and pressure. Geochim. Cosmochim. Acta.

[34] Zheng K., Liao J.L., Wang X.D. (2013). Raman spectroscopic study of the structural properties of CaO–MgO–SiO_2_–TiO_2_ slags. J. Non–Cryst. Solids.

[35] Shi Y., Huang W.M., Li J. (2020). Site–specific electrodeposition enables self–terminating growth of atomically dispersed metal catalysts. Nat. Commun..

[36] Pan W., Lian J. (1999). Thermodynamics of Ti in Cu–Ti alloy investigated by the EMF method. Mater. Sci. Eng., A.

[37] Pan W., Li R.L., Chen J. (2000). Thermodynamic properties of Ti in Ag–Ti alloys. Mat. Sci. Eng. A.

[38] Kong L.X., Xu J.J., Xu B.Q. (2016). Vapor–liquid phase equilibria of binary tin–antimony system in vacuum distillation: experimental investigation and calculation. Fluid Phase Equilib.

[39] Lu X.K., Bertei A., Finegan D.P. (2020). 3D microstructure design of lithium–ion battery electrodes assisted by X–ray nano–computed tomography and modelling. Nat. Commun..

[40] Xiong N., Tian Y., Yang B. (2018). Volatilization and condensation behaviours of Mg under vacuum. Vac.

[41] Pu Z.H., Han J.B., Li Y.F. (2018). Removal of arsenic from crude tin by vacuum distillation. Mater. Trans..

[42] US Geological Survey, 2022. 10.3133/mcs2022.

[43] Fan G.Q., Wang M., Dang J. (2021). A novel recycling approach for efficient extraction of titanium from high–titanium–bearing blast furnace slag. Waste Manage..

[44] Lv X., Lun Z., Yin J., Bai C. (2013). Carbothermic reduction of vanadium titanomagnetite by microwave irradiation and smelting behavior. ISIJ Int..

[45] Wang L., Liu W.Z., Hu J.P. (2018). Indirect mineral carbonation of titanium−bearing blast furnace slag coupled with recovery of TiO_2_ and Al_2_O_3_. Chin. J. Chem. Eng..

