# Supplementary material for: A recyclable method for titanium extraction and oxygen evolution from Ti−bearing slags
Source: Fundam Res. 2022 Dec 27;4(1):86–94. doi: 10.1016/j.fmre.2022.12.010 (PMC11197768; doi:10.1016/j.fmre.2022.12.010)
Supplement: Supplementary file 1 [file mmc1.docx]

**Supplementary Materials**

**A recyclable method for titanium extraction and oxygen evolution from Ti−bearing slags**

Zhenghao Pu^a^, Wei Wang^a,b,^*, Zhe Wang^a^, Mingyin Kou^b^, Yiwa Luo^b,c^, Jianbang Ge^b^, Xin Tao^a^, Mingyong Wang^a,c,^*, Shuqiang Jiao^a,b,c,^*

^a^ *State Key Laboratory of Advanced Metallurgy, University of Science and Technology Beijing, Beijing 100083, PR China.*

^b^ *School of Metallurgical and Ecological Engineering, University of Science and Technology Beijing, Beijing 100083, PR China.*

^c^ *Southern Marine Science and Engineering Guangdong Laboratory (Zhuhai), Zhuhai 519080, PR China.*

***Corresponding author**: wwang@ustb.edu.cn; mywang@ustb.edu.cn; sjiao@ustb.edu.cn

This PDF file includes:

The application evaluation of Ir anode
Properties of Ti−bearing slags
Application of liquid cathode in MOE

Abbreviations
Figures 1 to 18

Tables 1 to 6

Equations 1 to 14

References

**The application evaluation of Ir anode**

Thermogravimetric analysis−differential scanning calorimetry (TG–DSC) of IrO_2_ (Ar Condition) is analyzed at 25 to 1400℃. The IrO_2_ is relatively stable below 600 ℃ and will gradually decompose (lose weight) with the increase of temperature. When the temperature reaches ~930℃, IrO_2_ will decompose completely (Fig. 1) [1–3].

Cost–free Ti−bearing blast furnace slag (TB–slag, CaO–MgO–Al_2_O_3_–SiO_2_–TiO_2_, CaO: 26.46 wt%, MgO: 9.40 wt%, Al_2_O_3_: 15.35 wt%, SiO_2_: 26.76 wt%, TiO_2_: 22.03 wt%) is used as raw material, while cost–free electroslag (CaF_2_–Al_2_O_3_–CaO, CaF_2_: 60.00 wt%, Al_2_O_3_: 32.00 wt%, CaO: 8.00 wt%) and TB–slag are configured in equal proportions to form FTB–slag (CaF_2_–CaO–MgO–Al_2_O_3_–SiO_2_–TiO_2_, CaF_2_: 30.00 wt%, CaO: 17.23 wt%, MgO: 4.70 wt%, Al_2_O_3_: 23.67 wt%, SiO_2_: 13.38 wt%, TiO_2_: 11.02 wt%) and used together for testing to improve the conductivity of the electrolyte (Table 1, Fig. 2). With the addition of 30% CaF_2_, FTB–Slag has a greater alkalinity and conductivity with a lower hemispherical temperature than the original TB–slag.

Ir anodes are used to test chemical and electrochemical corrosion (1.5 A cm^–2^) at 1400 ℃. The corroded Ir anodes are analyzed using scanning electron microscopy (Fig. 3) [4,5]. The chemical corrosion rate of Ir in TB–Slag is significantly slower than that in FTB–Slag, and there is the ~20 μm oxide layer on the surface of Ir in both electrolytes, and calcium–rich phases are present in both oxide layers (Fig. 4). The elemental analysis results indicate that these phases belong to CaIrO_3_ or Ca_2_IrO_4_ (Fig. 5). However, Ir particles appear on the surface of Ir after electrochemical corrosion, and only tiny amount of CaIrO_3_ or Ca_2_IrO_4_ is present, which is attributed to the faster mass transfer. All of these Ir particles mentioned above may come from the decomposition of IrO_2_, CaIrO_3_ and Ca_2_IrO_4_.

**Properties of Ti−bearing slags**

The ion–molecule coexistence theory (IMCT) is used to calculate the activity of each oxide in TB–Slag. There are five oxides as CaO, MgO, Al_2_O_3_, SiO_2_, and TiO_2_ in TB–Slag [6–8]. With reference to the phase diagram between each oxide, TB–Slag consists of simple ions, simple molecules, and complex molecules as slag compositions, as follows:

Simple ions: Ca^2+^, Mg^2+^, O^2–^

Simple molecules: Al_2_O_3_, SiO_2_, TiO_2_

Complex molecules: 3CaO**·**SiO_2_, 2CaO**·**SiO_2_, CaO**·**SiO_2_, 3CaO**·**Al_2_O_3_, 12CaO**·**7Al_2_O_3_, CaO**·**Al_2_O_3_, CaO**·**2Al_2_O_3_, CaO**·**6Al_2_O_3_, 2MgO**·**SiO_2_, MgO**·**SiO_2_, MgO**·**Al_2_O_3_, 3Al_2_O_3_**·**2SiO_2_, CaO**·**TiO_2_, 3CaO**·**2TiO_2_, 4CaO**·**3TiO_2_, Al_2_O_3_**·**TiO_2_, MgO**·**TiO_2_, MgO**·**2TiO_2_, 2MgO**·**TiO_2_, 2CaO**·**Al_2_O_3_**·**SiO_2_, CaO**·**Al_2_O_3_**·**2SiO_2_, CaO**·**MgO**·**2SiO_2_, 2CaO**·**MgO**·**2SiO_2_, 3CaO**·**MgO**·**2SiO_2_, CaO**·**SiO_2_**·**TiO_2_

Based on the structure of the melt, the equilibrium constant equation of the reaction is established using the structural units of the slag according to IMCT, and finally, the activity calculation model of the slag is established using mass balance. Based on coexistence theory, ∑n is expressed as the total equilibrium moles of substances in each structural unit, and $N_{i}$ and $N_{\mathrm{ci}}$ are expressed as the activity of each oxide. Simple ions and molecules (Ca^2+^+O^2–^, Mg^2+^+O^2–^, Al_2_O_3_, SiO_2_ and TiO_2_) are represented by N1 to N5, and all complex molecules are displayed in Table 2. In addition, Table 2 presents information on the reaction equations of all complex molecules, where the activity of complex molecules is expressed by the equilibrium constants and the activity of simple molecules and ions.

According to the material balance, the following can be obtained:

$\sum_{i=1}^{25} N_{i}=1$ (1)

Meanwhile, according to the law of mass conservation, the masses of CaO, MgO, Al_2_O_3_, SiO_2_ and TiO_2_ in the slag should remain unchanged before and after the reaction. Eqs. 2–6 are obtained in turn, where b_1_, b_2_, b_3_, b_4_ and b_5_ are the total molar fractions of CaO, MgO, Al_2_O_3_, SiO_2_ and TiO_2_ before the reaction, respectively.

$b_{1}=\left( 0.5N_{1}+3N_{c1}+2N_{c2}+N_{c3}+{3N}_{c4}+{12N}_{c5}+N_{c6}+N_{c7}+N_{c13}+3N_{c14}+4N_{c15}+2N_{c20}+N_{c21}+N_{c22}+2N_{c23}+3N_{c24}+N_{c25} \right)\sum n$ (2)

$b_{2}=\left( 0.5N_{2}+2N_{c9}+N_{c10}+N_{c11}+N_{c17}+N_{c18}+2N_{c19}+N_{c22}+N_{c23}+N_{c24} \right)\sum n$ (3)

$b_{3}=\left( N_{3}+N_{c4}+7N_{c5}+N_{c6}+{2N}_{c7}+{6N}_{c8}+N_{c11}+{3N}_{c12}+N_{c16}+N_{c20}+N_{c21} \right)\sum n$ (4)

$b_{4}=\left( N_{4}+N_{c1}+N_{c2}+N_{c3}+N_{c9}+N_{c10}+2N_{c12}+N_{c20}+2N_{c21}+2N_{c22}+2N_{c23}+{2N}_{c24}+N_{c25} \right)\sum n$ (5)

$b_{5}=\left( N_{5}+N_{c13}+2N_{c14}+2N_{c15}+N_{c16}+N_{c17}+{2N}_{c18}+N_{c19}+N_{c25} \right)\sum n$ (6)

The nonlinear system of equations is solved by simultaneously associating Eq. 2 and Eqs. 3–6, and the activity of each oxide is obtained by Newton's iterative method.

The activities of the main oxides of TB–Slag is listed in Table 3. In TB–Slag, although the TiO_2_ content is lower than that of SiO_2_, the activity of TiO_2_ is twice as high as that of SiO_2_ because SiO_2_ is more acidic and binds more easily to CaO.

Due to the overly complex structure of TB–slag, TiO_2_–CaO (TC, TiO_2_: 78 wt%, CaO: 22 wt%) is prepared at 1600 ℃, and the Ti structure in TC is analyzed by synchrotron radiation after water quenching. The bond length and coordination number of Ti are analyzed by selecting TiO_2_ as the standard sample (Table 4), where CN is the coordination number, *R*(Å) is the distance between atoms, *σ*^2^(Å^2^) is the Debye–Waller factor to account for both thermal and structural disorders, Δ*E*_0_(eV) is the inner potential correction and the R factor indicates the goodness of the fit.

**Application of liquid cathode in MOE**

The galvanostatic electrolysis are performed using Ni, Pb, Sn and Sb as liquid cathodes, TB–Slag as the electrolyte and graphite as the anode at 1400 ℃ for 4 h (Figs. 9–12). There are almost no deposits in the electrolyzed Ni cathode, and the cathodic current efficiency is weak. The tiny deposits reveal the codeposition of Si and Ti, indicating that the Ni cathode cannot avoid the deposition of Si. A large amount of Si deposition occurs within the Pb cathode, while the Sn and Sb cathodes can effectively deposit Ti and the Sb cathode possesses a higher cathodic efficiency. It is noteworthy that Ca deposition also occurs within the Sb cathode. In summary, Sb is preferable used as the cathode for subsequent electrolysis coupled with the Ir anode.

To reveal the depolarization deposition mechanism of Ca and Ti within the Sb cathode after electrolysis, the activity of Ca and Ti in Sb–1Ca, Sb–1Ti and Sb–5Ti are measured by the electric potential method (Fig. 13), and the deposition potential is calculated according to the Nernst equation which combined with the determination of TiO_2_ and CaO activity in IMCT [9,10]. Sb alloys are used as working electrodes to assemble cells as follows:

$(Ar)Mo|Cr-\mathrm{Cr}_{2}O_{3}(s)|ZrO_{2}(MgO)|Sb-Ti-O(Melt)|(Ar)Mo$ (8)

$(Ar)Mo|Cr-\mathrm{Cr}_{2}O_{3}(s)|ZrO_{2}(MgO)|Sb-Ca-O(Melt)|(Ar)Mo$ (9)

According to the isothermal equation, it can be obtained:

$\Delta G_{m}=\Delta G_{m}^{\theta}+RTIn\frac{a_{\mathrm{Ti}_{in Sn}}}{P_{O_{2}\left( \mathrm{ref} \right)}^{\frac{1}{2}}}$ (10)

$\Delta G_{m}=-nEF$ (11)

$\Delta G_{m}^{\theta}=-RTInK=-RTIn\frac{a_{\mathrm{Ti}_{in Sn}}}{P_{O_{2}^{\theta}}^{\frac{1}{2}}}$ (12)

Where $\Delta G_{m}$ and $\Delta G_{m}^{\theta}$ are the Gibbs free energy of the total reaction and the self–Booth free energy under standard conditions, and $P_{O_{2}\left( \mathrm{ref} \right)}^{\frac{1}{2}}$ and $P_{O_{2}^{\theta}}^{\frac{1}{2}}$ are the partial pressure of the reaction O_2_ at the reference electrode and the equilibrium reaction O_2_ partial pressure at standard conditions, respectively. R is the gas constant, T is the reaction temperature, and F is the Faraday constant. The joint Eqs. 10–12 can be obtained as follows:

$EMF=\frac{\mathrm{RT}}{F}\mathrm{In}\frac{P_{O_{2}}^{\frac{1}{4}}\left( \mathrm{ref} \right)}{P_{O_{2}^{\theta}}^{\frac{1}{4}}}$ (13)

The electronic conductance needs to be corrected because of measurement under high temperature conditions:

$EMF=\frac{\mathrm{RT}}{F}\mathrm{In}\frac{P_{e}^{\frac{1}{4}}+P_{O_{2}}^{\frac{1}{4}}\left( \mathrm{ref} \right)}{P_{e}^{\frac{1}{4}}+P_{O_{2}^{\theta}}^{\frac{1}{4}}}$ (14)

Where $P_{e}$ is the electronically conducting characteristic O_2_ partial pressure of the reference electrode [11,12]. The activities of alloys can be calculated from Eq. 14. Meanwhile, the activities in Sb–Ca alloy are calculated in the same way as the Sb–Ti alloy, and the calculated activity data are presented in Table 5.

Sb is used as the cathode and Ir as the anode, and galvanostatic electrolysis is performed in TB–Slag at 1400 °C for 4 h at 0.75, 1.5 and 2.25 A cm^–2^, respectively (Figs. 14–18). The results of ICP analysis of the cathode after electrolysis are presented in Table 6.

*Abbreviations:* **MOE**: molten oxide electrolysis; **TB−Slag**: Ti−bearing blast furnace slag; **FTB−Slag**: Ti−bearing blast furnace slag with added electroslag cosolvent; **DFT**: density function theory; **VASP**: Vienna Ab−initio simulation package; **DOS:** density of states; **OER**: oxygen evolution reaction; **XANES**: X−ray near−edge spectroscopy; **TC**: TiO_2_−CaO; **CV**: cyclic voltammetry.

**References**

1. K.T. Jacob, T.H. Okabe, T. Uda, Y. Waseda, Solid–state cells with buffer electrodes for the measurement of thermodynamic properties of IrO_2_, CaIrO_3_, Ca_2_IrO_4_, Ca_4_IrO_6_, J. Electrochem. Soc. 146 (1999) 1854–1861.
2. C.L. Mcdaniel, S.J. Schneuder, Phase relations in the CaO–IrO_2_–Ir system in air, J. Solid. State. Chem. 4 (1972) 275–280.
3. W.B. Bell, M. Tagami, Study of Gaseous Oxides, Chloride, and Oxychloride of Iridium, J. Phys. Chem. 70 (1966) 640–646.
4. H.J. Kim, J. Paramore, A. Allanore, D.R. Sadoway, Stability of iridium anode in molten oxide electrolysis for ironmaking: influence of slag basicity, ECS Trans. 33 (2010) 219–230.
5. D.H. Wang, A.J. Gmitter, D.R. Sadoway, Production of oxygen gas and liquid metal by electrochemical decomposition of molten iron oxide, J. Electrochem., Soc. 158 (2011) E51–E54.
6. S.C. Duan, X.L. Guo, H.J. Guo, J. Guo, A manganese distribution prediction model for CaO–SiO_2_–FeO–MgO–MnO–Al_2_O_3_ slags based on IMCT, Ironmak. Steelmak. 44 (2016) 168–184.
7. C.B. Shi, X.M. Yang, J.S. Jiao, C. Li, H.J. Guo, A Sulphide Capacity Prediction Model of CaO–SiO_2_–MgO–Al_2_O_3_ Ironmaking Slags Based on the Ion and Molecule Coexistence Theory, ISIJ Int. 50 (2010) 1362–1372.
8. Y. Zhou, R. Zhu, H.Y. Wang, H.J. Zhang, Effect of various components on the distribution of phosphorus in CaO–FeO–MgO–SiO_2_–MnO–TiO_2_–V_2_O_5_–P_2_O_5_ slag based on IMCT, Ironmak. Steelmak. 48 (2020) 570–571.
9. N.D. Smith, T. Lichtenstein, J. Gesualdi, K. Kumar, H.J. Kim, Thermodynamic properties of strontium–bismuth alloys determined by electromotive force measurements, Electrochim. Acta 225 (2017) 584–591.
10. L.X. Kong, J.J. Xu, B.Q. Xu, et al., Vapor–liquid phase equilibria of binary tin–antimony system in vacuum distillation: Experimental investigation and calculation, Fluid Phase Equilib. 415 (2016) 176–183.
11. W. Pan, J. Lian, Thermodynamics of Ti in Cu–Ti alloy investigated by the EMF method, Mater. Sci. Eng., A 269 (1999) 104–110.
12. W. Pan, R.L. Li, J. Chen, R.F. Sun, J. Lian, Thermodynamic properties of Ti in Ag–Ti alloys, Mat. Sci. Eng. A A287 (2000) 72–77.

Figures

**
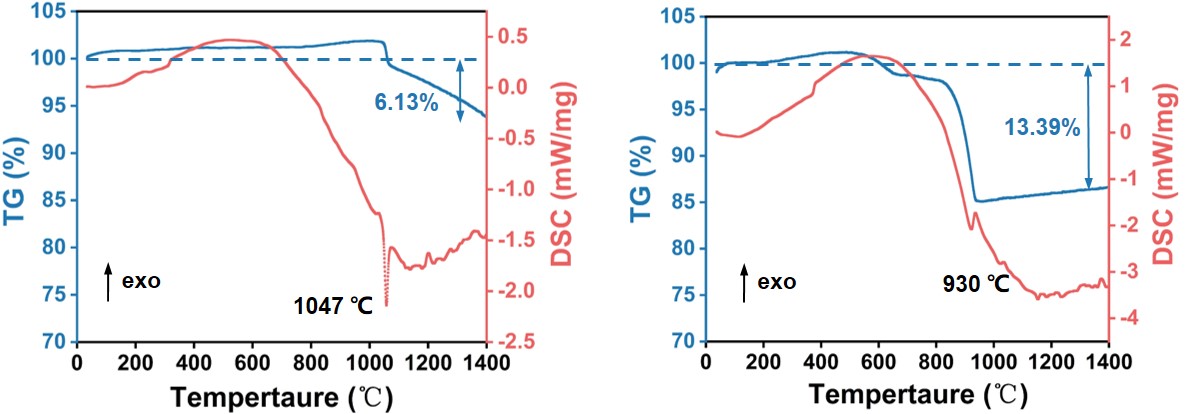
**

Fig. 1 Thermogravimetric analysis−differential scanning calorimetry (TG−DSC) analysis of IrO_2_ from 25 to 1400 °C (Ar).


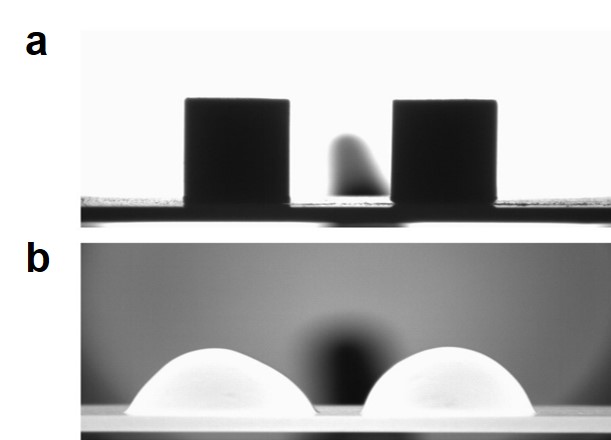


Fig. 2 Hemispherical temperature determination of titanium−bearing blast furnace slag (a) before melting (b) after melting.

**
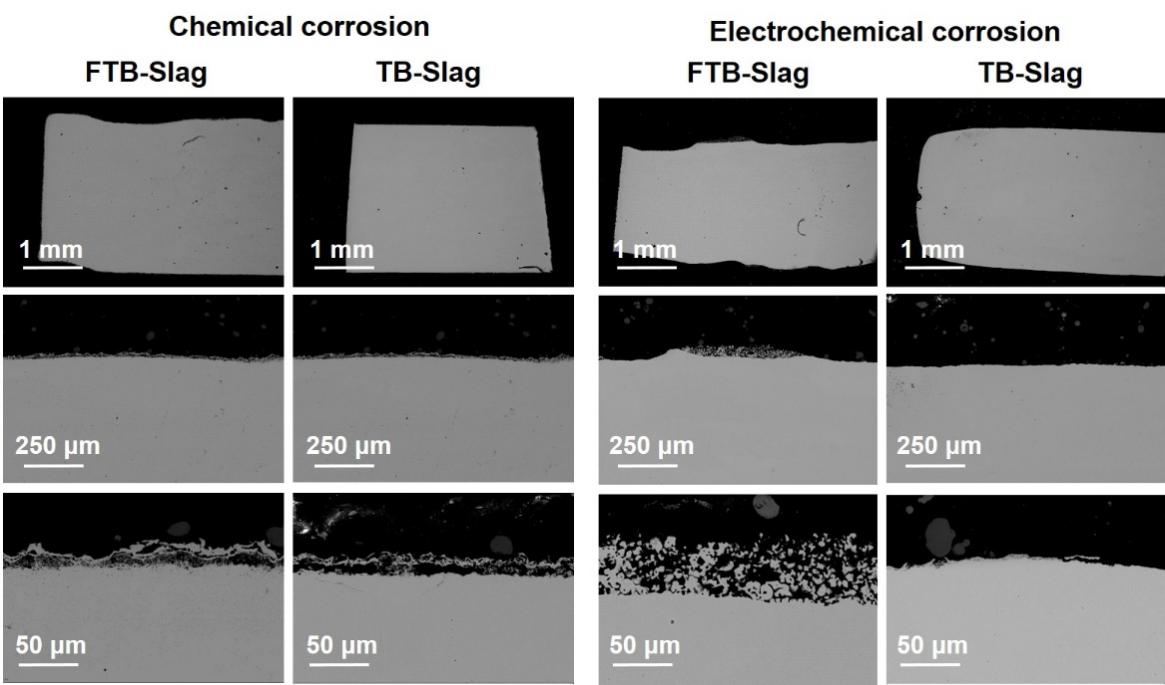
**

Fig. 3 SEM images of Ir anodic chemical corrosion and electrochemical corrosion (1.5 A cm^−2^) for 4 h.


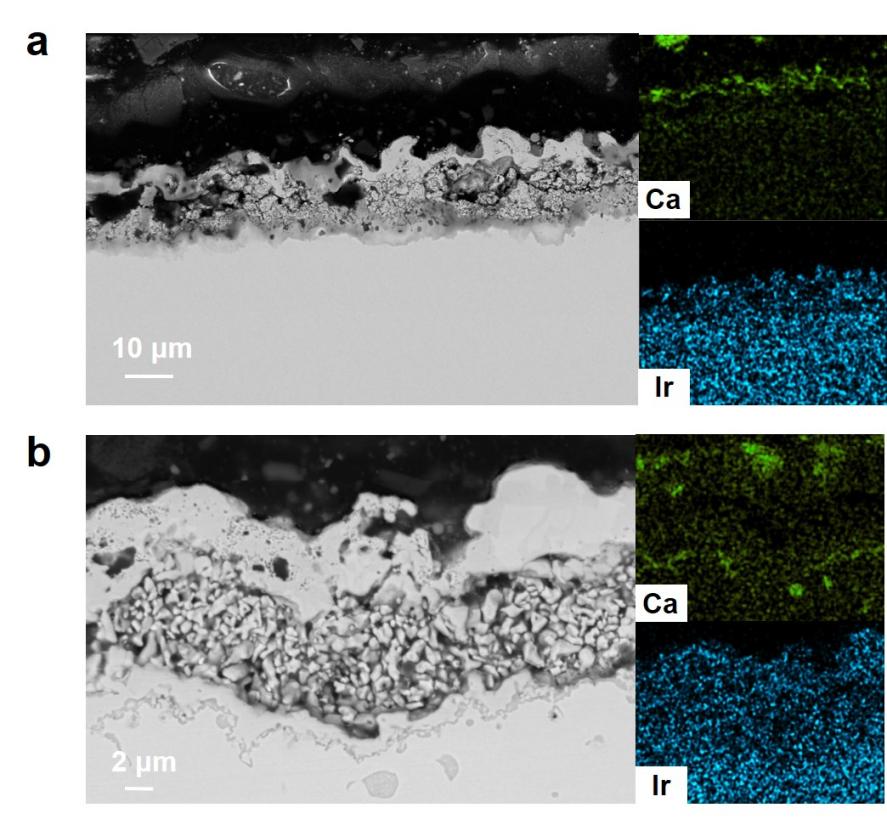


Fig. 4 EDS analysis of chemical corrosion at the Ir interface: (a) TB–slag and (b) FTB–slag.


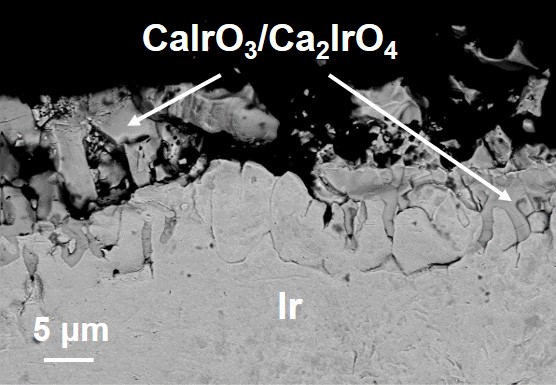


Fig. 5 SEM image of Ir anode interface oxide layer.


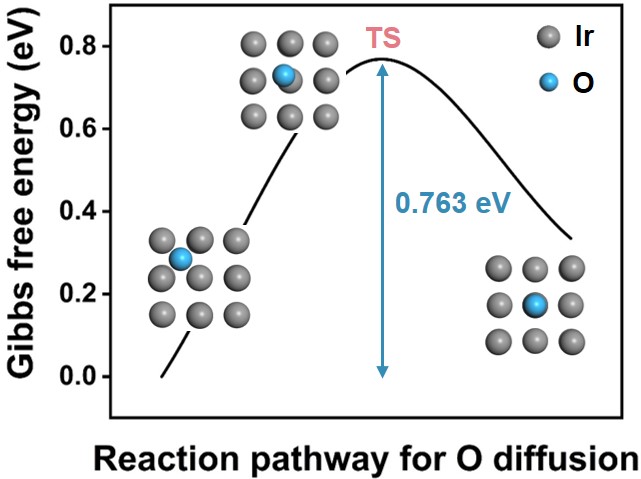


Fig. 6 Calculation of the Gibbs free energy change of O diffusion at the Ir interfacial.


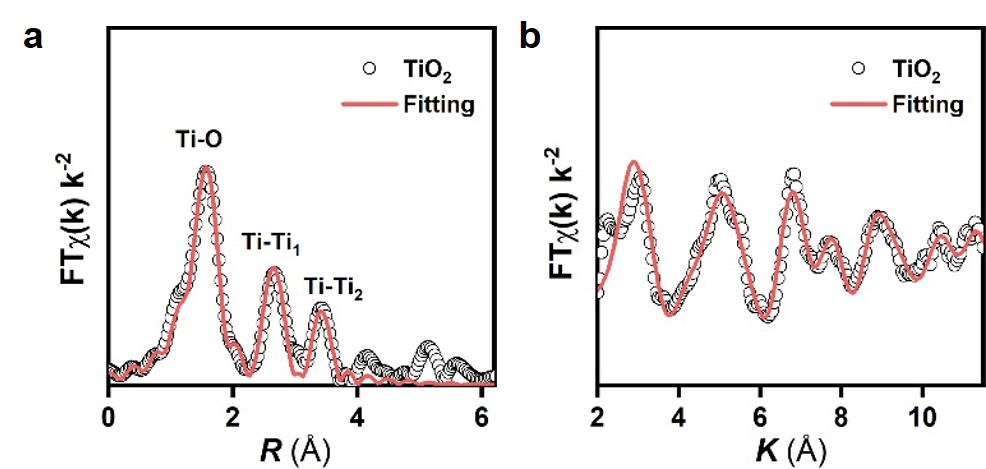


Fig. 7 (a) R−space fit of TiO_2_. (b) K−space fit of TiO_2_.


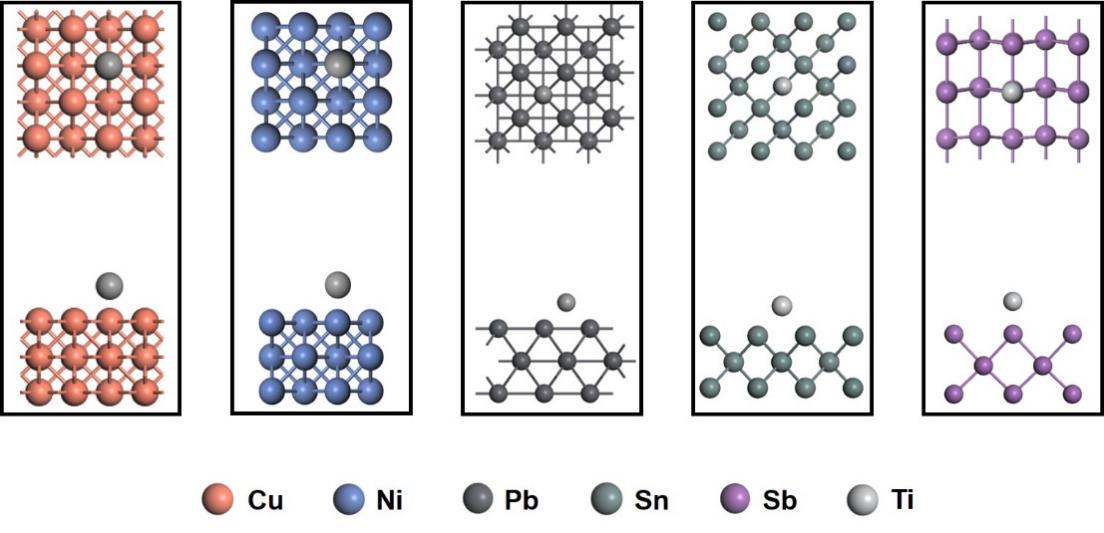


Fig. 8 Model for simulation calculations


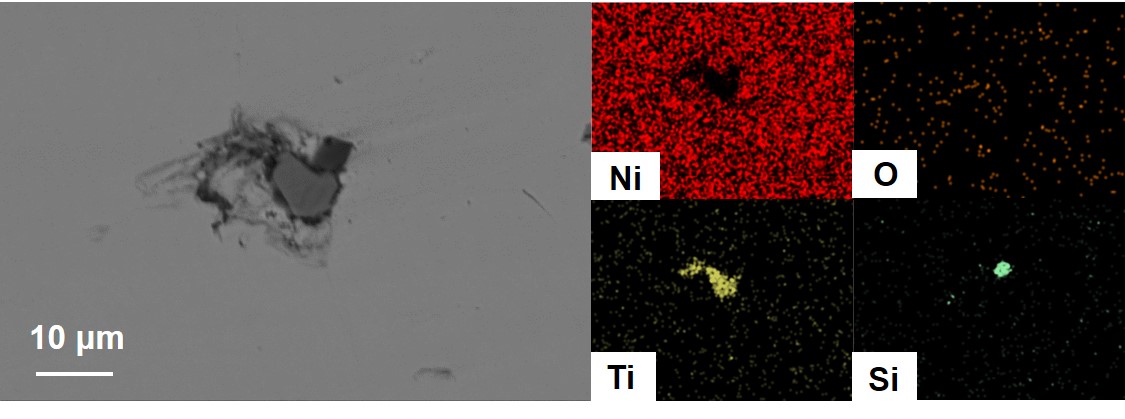


Fig. 9 SEM−EDS images of the liquid Ni cathode after electrolysis.


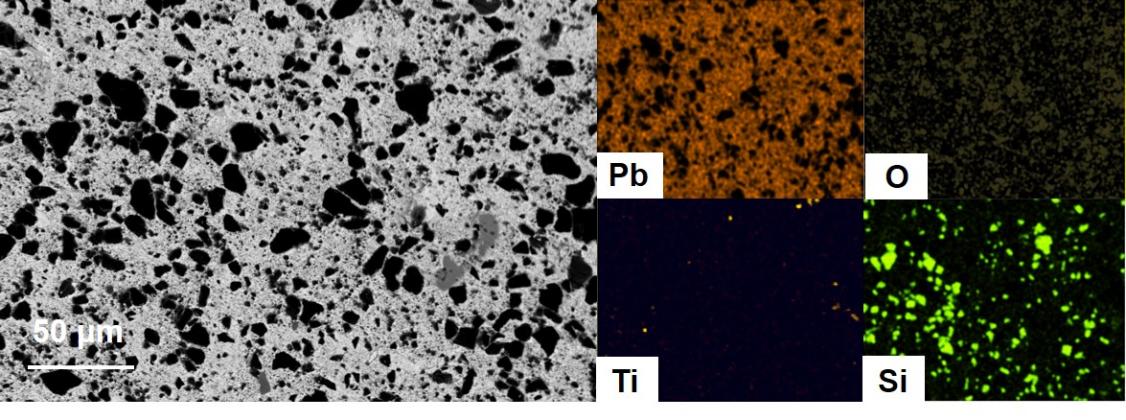


Fig. 10 SEM−EDS images of the liquid Pb cathode after electrolysis.


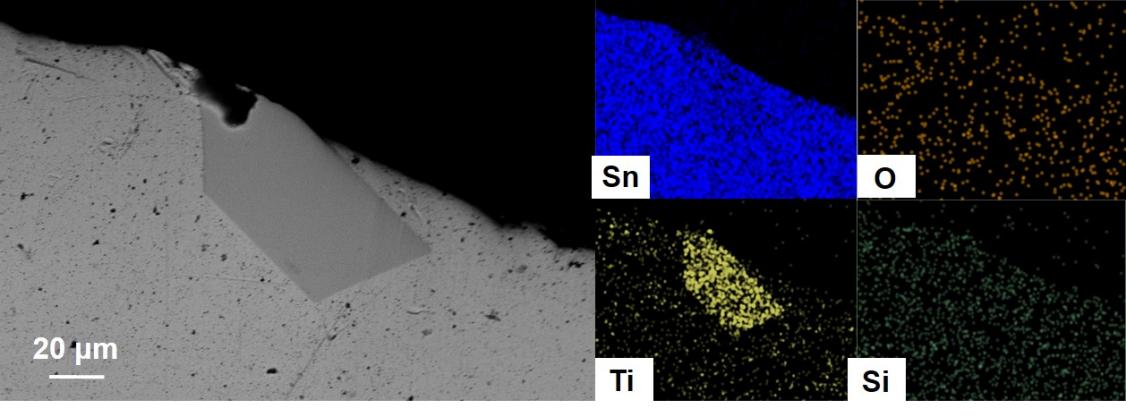


Fig. 11 SEM−EDS images of the liquid Sn cathode after electrolysis.


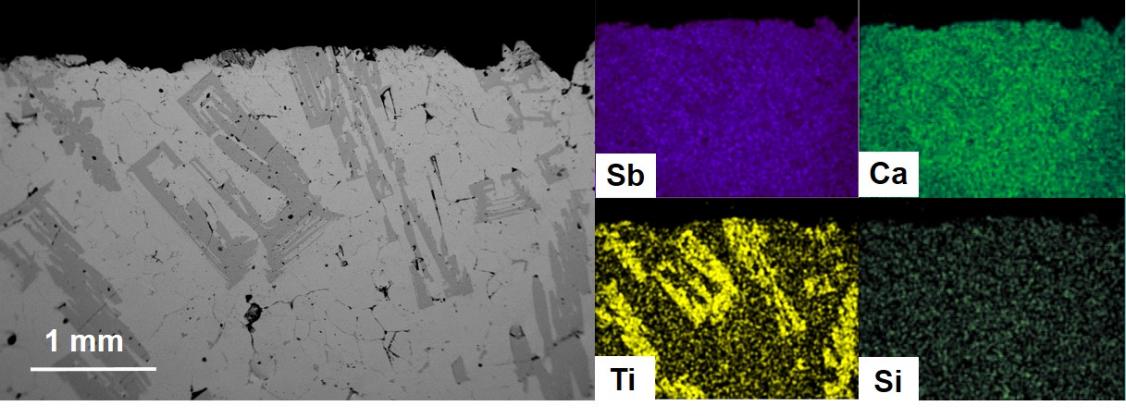


Fig. 12 SEM−EDS images of the liquid Sb cathode after electrolysis.


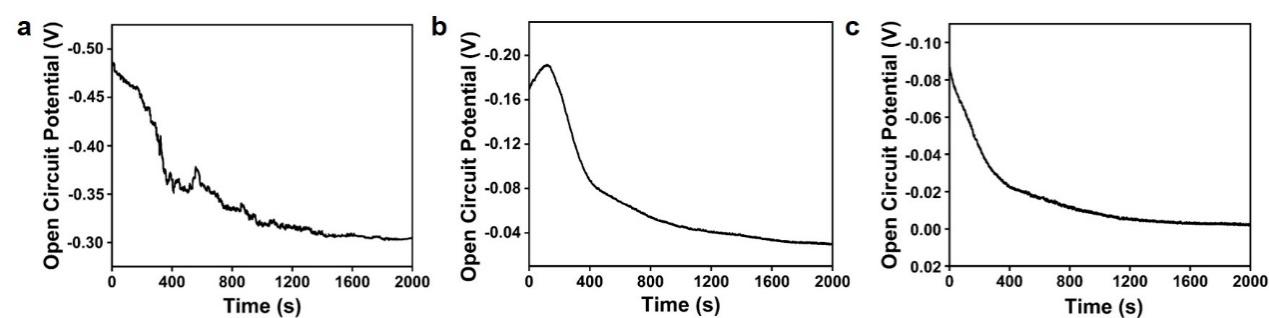


Fig. 13 Open circuit potential measurement (a) Sb−1Ca, (b) Sb−1Ti, and (c) Sb−5Ti.


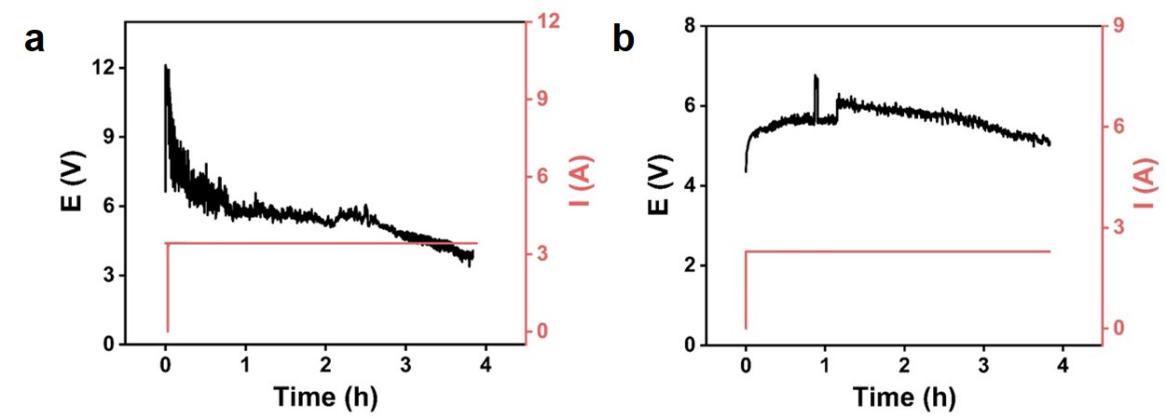


Fig. 14 E–I curve of galvanostatic electrolysis at 1400 ℃ for 4 h with (a) 2.25 A cm^−2^ and (b) 1.5 A cm^−2^ for TB–Slag.


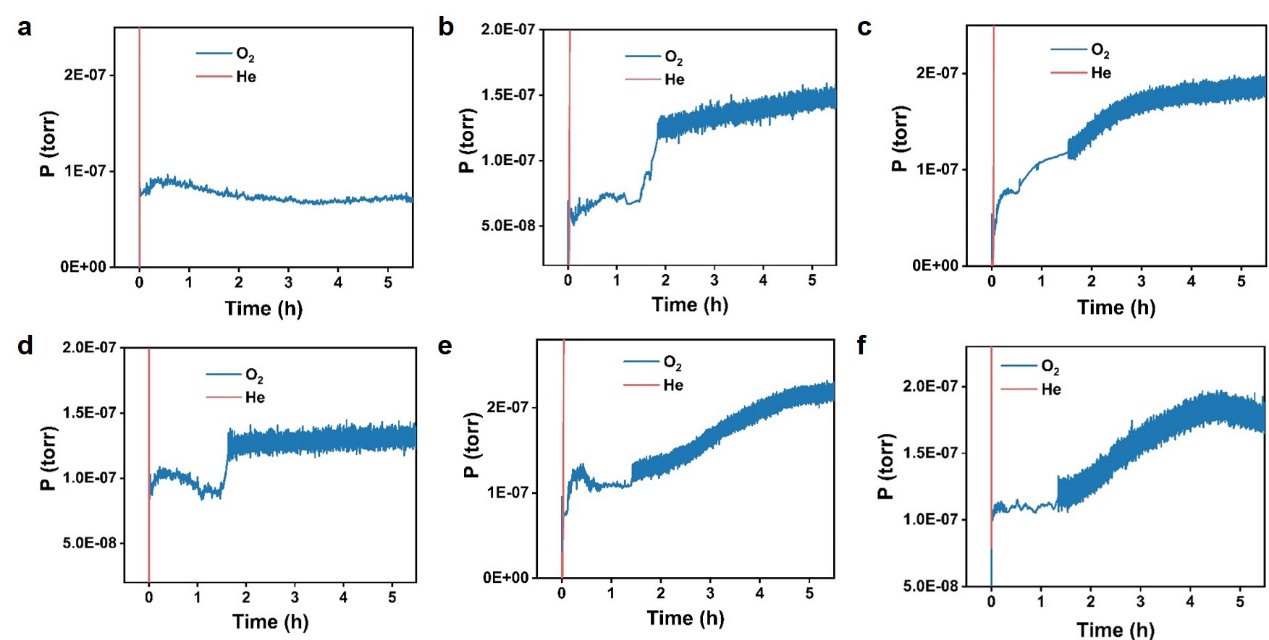


Fig. 15 Online gas monitoring analysis of the Sb cathode electrolysis process at 1400 ℃ for 4 h with (a) 0.75 A cm^−2^, (b) 1.5 A cm^−2^ and (c) 2.25 A cm^−2^ for TB–Slag.


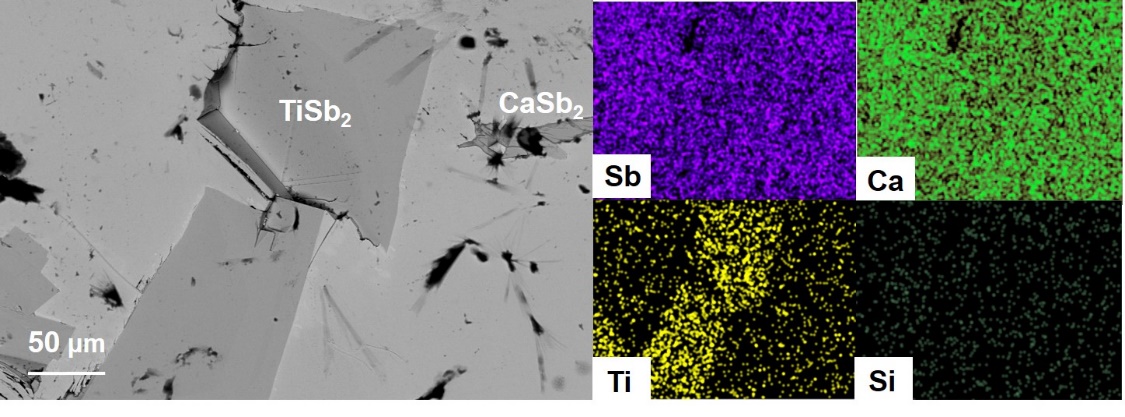


Fig. 16 SEM–ESD composition analysis of the Sb cathode after electrolysis.


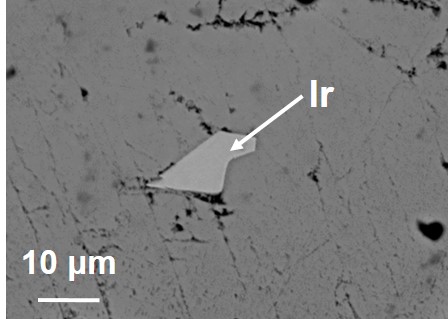


Fig. 17 Ir deposition inside the Sb cathode after electrolysis.


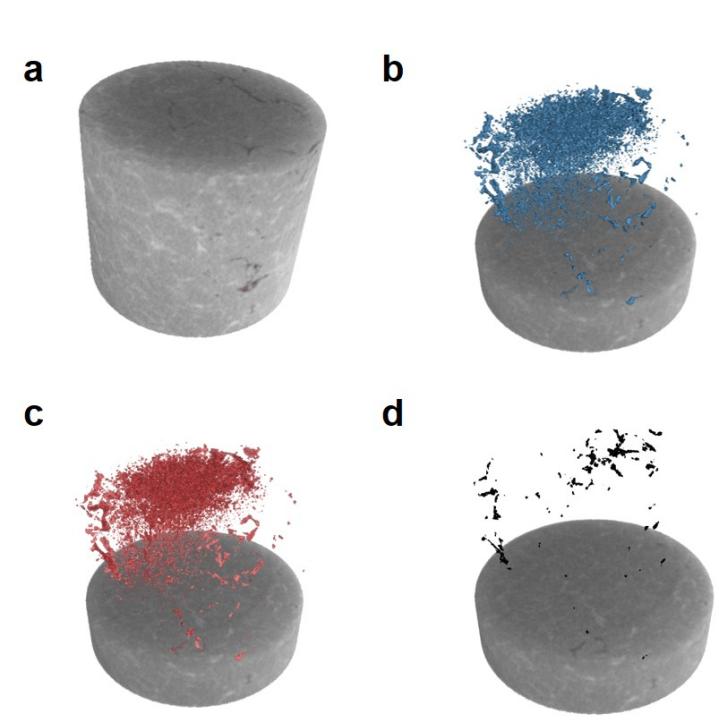


Fig. 18 (a) CT reconstruction analysis of the Sb cathode after electrolysis in TB−Slag (1400 ℃, 2.25 A cm^−2^) (b) Reconstruction image of rich Ca phase. (c) Reconstruction image of rich Ti phase. (d) Reconstruction image of the pores.

Tables

Table 1. Electrical conductivity of metallurgical slag.

| Slag | CaO | MgO | Al_2_O_3_ | SiO_2_ | TiO_2_ | CaF_2_ | Alkalinity | Electrical conductivity/S cm^–1^ | Hemispherical temperature/℃ |
| --- | --- | --- | --- | --- | --- | --- | --- | --- | --- |
| TB–Slag | 26.46 | 9.4 | 15.35 | 26.76 | 22.03 | — | 0.99 | 0.2 | 1257.6 |
| FTB–Slag | 17.23 | 4.7 | 23.67 | 13.38 | 11.02 | 30 | 1.29 | 0.67 | 1172.4 |

Table 2. Chemical reaction, Gibbs free energy and activity expression of complex molecules for TB–Slag.

| Reaction equations | $\Delta G^{\theta}/\left( J {mol}^{-1} \right)$ | $N_{\mathrm{ci}}$ |
| --- | --- | --- |
| 3(Ca^2+^+O^2–^)+(SiO_2_)=(3CaO·SiO_2_) | –93366–23.03T | N_c1_=K_c1_N_1_^3^N_4_ |
| 2(Ca^2+^+O^2–^)+(SiO_2_)=(2CaO·SiO_2_) | –160431+4.106T | N_c2_=K_c2_N_1_^2^N_4_ |
| (Ca^2+^+O^2–^)+(SiO_2_)=(CaO·SiO_2_) | –81416–10.498T | N_c3_=K_c3_N_1_N_4_ |
| 3(Ca^2+^+O^2–^)+(Al_2_O_3_)=(3CaO·Al_2_O_3_) | –21771.36–29.31T | N_c4_=K_c4_N_1_^3^N_3_ |
| 12(Ca^2+^+O^2–^)+7(Al_2_O_3_)=(12CaO·7Al_2_O_3_) | –103240–311.1T | N_c5_=K_c5_N_1_^12^N_3_^7^ |
| (Ca^2+^+O^2–^)+(Al_2_O_3_)=(CaO·Al_2_O_3_) | –23027.4–18.84T | N_c6_=K_c6_N_1_N_3_ |
| (Ca^2+^+O^2–^)+2(Al_2_O_3_)=(CaO·2Al_2_O_3_) | –16747.2–25.4T | N_c7_=K_c7_N_1_N_3_^2^ |
| (Ca^2+^+O^2–^)+6(Al_2_O_3_)=(CaO·6Al_2_O_3_) | –22608.72–31.82T | N_c8_=K_c8_N_1_N_3_^6^ |
| 2(Mg^2+^+O^2–^)+(SiO_2_)=(2MgO·SiO_2_) | –63220+1.884T | N_c9_=K_c9_N_2_^2^N_4_ |
| (Mg^2+^+O^2–^)+(SiO_2_)=(MgO·SiO_2_) | –36452–11.675T | N_c10_=K_c10_N_2_N_4_ |
| (Mg^2+^+O^2–^)+(Al_2_O_3_)=(MgO·Al_2_O_3_) | –35530–2.09T | N_c11_=K_c11_N_2_N_3_ |
| 3(Al_2_O_3_)+2(SiO_2_) =(3Al_2_O_3_·2SiO_2_) | –4354.27–10.467T | N_c12_=K_c12_N_3_^3^N_4_^2^ |
| (Ca^2+^+O^2–^)+(TiO_2_)=(CaO·TiO_2_) | –79900–3.35T | N_c13_=K_c13_N_1_N_5_ |
| 3(Ca^2+^+O^2–^)+2(TiO_2_)=(3CaO·2TiO_2_) | –207100–11.51T | N_c14_=K_c14_N_1_^3^N_5_^2^ |
| 4(Ca^2+^+O^2–^)+3(TiO_2_)=(4CaO·3TiO_2_) | –292900–17.57T | N_c15_=K_c15_N_1_^4^N_5_^3^ |
| (Al_2_O_3_)+(TiO_2_) =(Al_2_O_3_·TiO_2_) | –5439–8.351T | N_c16_=K_c16_N_3_N_5_ |
| (Mg^2+^+O^2–^)+(TiO_2_)=(MgO·TiO_2_) | –25104+2.804T | N_c17_=K_c17_N_2_N_5_ |
| (Mg^2+^+O^2–^)+2(TiO_2_)=(MgO·2TiO_2_) | –18619–7.99T | N_c18_=K_c18_N_2_N_5_^2^ |
| 2(Mg^2+^+O^2–^)+(TiO_2_)=(2MgO·TiO_2_) | –17154–10.878T | N_c19_=K_c19_N_2_^2^N_5_ |
| 2(Ca^2+^+O^2–^)+(Al_2_O_3_)+(SiO_2_)=(2CaO·Al_2_O_3_·SiO_2_) | –61964.64–60.29T | N_c20_=K_c20_N_1_^2^N_3_ N_4_ |
| (Ca^2+^+O^2–^)+(Al_2_O_3_)+(2SiO_2_)=(CaO·Al_2_O_3_·2SiO_2_) | –138968.8+17.15T | N_c21_=K_c21_N_1_N_3_N_4_^2^ |
| (Ca^2+^+O^2–^)+(Mg^2+^+O^2–^)+(2SiO_2_)=(CaO·MgO·2SiO_2_) | –80387–51.916T | N_c22_=K_c22_N_1_N_2_N_4_^2^ |
| 2(Ca^2+^+O^2–^)+(Mg^2+^+O^2–^)+(2SiO_2_)=(2CaO·MgO·2SiO_2_) | –73688–63.69T | N_c23_=K_c23_N_1_^2^N_2_N_4_^2^ |
| 3(Ca^2+^+O^2–^)+(Mg^2+^+O^2–^)+(2SiO_2_)=(3CaO·MgO·2SiO_2_) | –315469+24.786T | N_c24_=K_c24_N_1_^3^N_2_N_4_^2^ |
| (Ca^2+^+O^2–^)+(SiO_2_)+(TiO_2_)=(CaO·SiO_2_·TiO_2_) | –122591.2+10.88T | N_c25_=K_c25_N_1_N_4_N_5_ |

Table 3. Activity of each major oxide in TB–Slag.

| Oxides | Temperature/℃ | TiO_2_ | SiO_2_ | CaO | MgO | Al_2_O_3_ |
| --- | --- | --- | --- | --- | --- | --- |
| TB–Slag | 1400 | 0.0866 | 0.0420 | 0.0020 | 0.0236 | 0.0755 |

Table 4. EXAFS fitting parameters at the Ti K–edge for various samples.

| Sample | Shell | *CN^a^* | *R*(Å)*^b^* | *σ*^2^(Å^2^)*^c^* | Δ*E*_0_(eV)*^d^* | *R* factor |
| --- | --- | --- | --- | --- | --- | --- |
| TiO_2_ | Ti–O | 6* | 1.94±0.01 | 0.0022±0.0012 | 9.2±1.3 | 0.0162 |
|  | Ti–Ti_1_ | 4* | 3.01±0.01 | 0.0027±0.0012 | 3.3±2.3 |  |
|  | Ti–Ti_2_ | 4* | 3.82±0.02 | 0.0025±0.0018 | 3.6±1.7 |  |
| TC | Ti–O | 5.1±0.9 | 1.93±0.02 | 0.0051±0.0024 | 9.3±1.3 | 0.0179 |
|  | Ti–Ti_1_ | 3.1±1.1 | 3.00±0.01 | 0.0081±0.0070 | –6.7±2.3 |  |
|  | Ti–Ti_2_ | 2.6±1.2 | 3.80±0.01 | 0.0050±0.0041 | –6.3±1.0 |  |

Table 5. The thermodynamic data for Sb alloys at 1400 ℃.

| Initial composition | $X_{i}$ | $P_{O_{2}}(ref)({10}^{-10})$ | $P_{O_{2}}({10}^{-10})$ | $a_{X}({10}^{-5})$ |
| --- | --- | --- | --- | --- |
| Sb–1Ti | 0.89 | 2.49 | 5.92 | 1.15 |
| Sb–5Ti | 4.98 | 2.49 | 2.64 | 1.72 |
| Sb–1Ca | 0.71 | 2.49 | $1.{2\times10}^{4}$ | ${2\times10}^{-4}$ |

Table 6. Experimental results after electrolysis under different current densities in TB–slag of the Sb cathode.

| Slag | Current density  (A cm^–2^) | Ti content (*wt*%) | Ca content (*wt*%) | Si content (*wt*%) |
| --- | --- | --- | --- | --- |
| TB–Slag | 0.05 | – | – | – |
|  | 0.1 | 0.10 | 0.08 | 0.0017 |
|  | 0.15 | 0.51 | 0.44 | – |
